# Analyzing Runs of Homozygosity Reveals Patterns of Selection in German Brown Cattle

**DOI:** 10.3390/genes15081051

**Published:** 2024-08-09

**Authors:** Anna Wirth, Jürgen Duda, Reiner Emmerling, Kay-Uwe Götz, Franz Birkenmaier, Ottmar Distl

**Affiliations:** 1 Institute of Animal Breeding and Genetics, University of Veterinary Medicine Hannover (Foundation), 30559 Hannover, Germany; anna.wirth@tiho-hannover.de; 2 Landeskuratorium der Erzeugerringe für Tierische Veredelung in Bayern e.V. (LKV), 80687 München, Germany; juergen.duda@lkv.bayern.de; 3 Bavarian State Research Center for Agriculture, Institute of Animal Breeding, 85586 Poing-Grub, Germany; reiner.emmerling@lfl.bayern.de (R.E.); kay-uwe.goetz@lfl.bayern.de (K.-U.G.); 4 Amt für Ernährung, Landwirtschaft und Forsten, 87439 Kempten, Germany; franz.birkenmaier@aelf-ke.bayern.de

**Keywords:** runs of homozygosity islands, US Brown Swiss, original brown, survival, genomic inbreeding, effective population size

## Abstract

An increasing trend in ancestral and classical inbreeding coefficients as well as inbreeding depression for longevity were found in the German Brown population. In addition, the proportion of US Brown Swiss genes is steadily increasing in German Browns. Therefore, the aim of the present study was to analyze the presence and genomic localization of runs of homozygosity (ROH) in order to evaluate their associations with the proportion of US Brown Swiss genes and survival rates of cows to higher lactations. Genotype data were sampled in 2364 German Browns from 258 herds. The final data set included 49,693 autosomal SNPs. We identified on average 35.996 ± 7.498 ROH per individual with a mean length of 8.323 ± 1.181 Mb. The genomic inbreeding coefficient F_ROH_ was 0.122 ± 0.032 and it decreased to 0.074, 0.031 and 0.006, when genomic homozygous segments > 8 Mb (F_ROH>8_), >16 Mb (F_ROH>16_) and >32 Mb (F_ROH>32_) were considered. New inbreeding showed the highest correlation with F_ROH>32_, whereas ancestral inbreeding coefficients had the lowest correlations with F_ROH>32_. The correlation between the classical inbreeding coefficient and F_ROH_ was 0.572. We found significantly lower F_ROH_, F_ROH>4_, F_ROH>8_ and F_IS_ for US Brown Swiss proportions <60% compared to >80%. Cows surviving to the 2nd, 4th, 6th, 8th, and 10th lactation had lower genomic inbreeding for F_ROH_ and up to F_ROH>32_, which was due to a lower number of ROH and a shorter average length of ROH. The strongest ROH island and consensus ROH shared by 50% of the animals was found on BTA 6 at 85–88 Mb. The genes located in this genomic region were associated with longevity (*NPFFR2* and *ADAMTS3*), udder health and morphology (*SLC4A4*, *NPFFR2*, GC and *RASSF6)*, milk production, milk protein percentage, coagulation properties of milk and milking speed (*CSN3*). On BTA 2, a ROH island was detected only in animals with <60% US Brown Swiss genes. Genes within this region are predominantly important for dual-purpose cattle breeds including Original Browns. For cows reaching more than 9 lactations, an exclusive ROH island was identified on BTA 7 with genes assumed to be associated with longevity. The analysis indicated that genomic homozygous regions important for Original Browns are still present and also ROH containing genes affecting longevity may have been identified. The breeding of German Browns should prevent any further increase in genomic inbreeding and run a breeding program with balanced weights on production, robustness and longevity.

## 1. Introduction

German Brown cattle have been developed in the Alpine region in South Germany as a dual-purpose breed for butter and cheese production as well as for fattening of surplus calves to produce valuable carcasses. This breed is characterized by good quality legs and claws, so that the cows can withstand the harsh environmental conditions in this mountainous region. Their robust health and high fertility enable the cows to have high lifetime production and a long life [1]. The breeding history of the German Brown and other European Brown populations is shaped by the introgression of US Brown Swiss bulls over the last 50 years [2]. With increasing US Brown Swiss blood proportions, milk performance increased, but longevity and lifetime performance were highest with US Brown Swiss blood proportions of 41–50% [3,4]. The ancestral (inbred common ancestors in the pedigree) and new inbreeding (inbred animal in the pedigree for the first time) coefficients from pedigree data in the German Brown population show increasing trends, but the effects of inbreeding depression on longevity and lifetime production were counterbalanced by positive heterosis effects [5,6]. A further increase in inbreeding is expected to reduce the positive heterosis effects and negatively influence the lifetime production and cow survival rates [6]. A decrease in genetic diversity and effective population size can have negative effects on adaptability to a changing environment [5].

An evaluation of inbreeding and its effects on the genome architecture in the actual German Brown population can be obtained from genomic data. A popular method used in genomic studies is the analysis of runs of homozygosity (ROH) [7,8,9,10,11,12,13,14,15]. They are assumed to be the result of the transmission of identical haplotypes from parents to offspring and are considered as an indicator of the degree of autozygosity. Since recombination during meiosis leads to shortening of ROH segments, it is assumed that short ROH are the result of inbreeding and selection [16]. The distribution and frequency of ROH of different lengths is population-specific and depends on the selection intensity and direction to which the respective population was exposed in its breeding history [7,15]. Furthermore, genomic regions that contain a high percentage of ROH (ROH islands) are regions subject to strong selection, as previously shown by overlapping the results of the analysis of selection signatures and ROH islands [16,17], and they may harbor genes involved in phenotypic characteristics of a breed [12,18,19]. Further analysis of these regions can therefore shed light on the nature of selection within a breed and reveal loci associated with economically important traits [20].

For European Brown Swiss populations, genome wide association studies (GWAS) have identified regions associated with production, udder morphology, fertility, calving and birth, body conformation and carcass traits based on different numbers of animals and SNPs, using deregressed breeding values of bulls as phenotypes for the analysis (Supplementary Material Tables S1 and S2) [21,22,23,24,25,26,27]. Few studies have distinguished between Original Brown breeds and modern Brown Swiss breeds that contain a proportion of US Brown Swiss blood [25,27], but it has been shown that modern Brown populations are one of the most differentiated breeds, with a strong genetic divergence from the Original Brown breeds [28]. It can therefore be expected that different selection objectives between Original Brown breeds and modern Brown breeds have targeted different genomic regions, resulting in different genomic localizations and the sizes of the ROH and ROH islands. Studies of ROH islands and selection signatures in Brown Swiss populations have been conducted on US Brown Swiss, Italian, German, Austrian and Swiss Brown and Original Brown, mostly based on medium density SNP data, with 27 K–62 K SNPs (Supplementary Material Table S3). 

Nevertheless, differences between Brown Swiss and Original Brown populations were evident between and within studies. In Brown Swiss populations, the region on BTA 6 between 80–95 Mb and the region on BTA 5 between 75 and 80 Mb were frequently identified as ROH islands or selection signatures (Supplementary Material Table S4), harboring genes associated with milk production traits, udder health and longevity. The QTL around 90 Mb was identified as a target of ongoing selection in Brown Swiss [23], confirming the strong focus of selection on production and udder conformation in Brown Swiss populations (Supplementary Material Table S4). In contrast, the selection pressure in Original Brown populations was more on BTA 11, as ROH islands [18] and selection signatures [29,30,31] were detected between 65 and 73 Mb with genes related to fertility, fat deposition, meat quality, adaption and immune response, and on BTA 26 between 21 and 23 Mb with genes such as *FGF8*, associated with meat and carcass quality (Supplementary Material Table S5). A QTL for body size and leg conformation was already found in this region in Brown Swiss [23].

To our knowledge, no study to date has analyzed the ROH structure in a large number of randomly selected German Brown cows and bulls with special attention to the breed proportion of US Brown Swiss and the association with survival as an important functional trait for this breed. Therefore, the aim of this study was to characterize the ROH structure and ROH-based inbreeding coefficients to evaluate the breeding history in the context of selection and inbreeding in 2364 randomly selected German Brown cattle and to compare these results with previous reports. In addition, we aimed to analyze common ROH and ROH islands to show the effects of selection on the genomic architecture in the German Brown. To better understand the influence of US Brown Swiss sires in German Brown cattle, we also performed these analyses for different classes of US Brown Swiss breed proportions and survival rates to higher lactation numbers. 

## 2. Materials and Methods

### 2.1. Animals

Genotype data of 2364 German Browns, including 74 German Brown bulls, were provided by the Allgäuer Herdebuchgesellschaft (AHG), Kempten, Germany. The cows were from 258 herds that participated in the genotyping program of the AHG. These herds were randomly selected within the cattle breeding organization AHG (Supplementary Material Table S6), and within these herds, cows having survived five lactations and their younger herdmates were randomly selected. In order to achieve an even distribution according to relationship, the sire and maternal grandsire were taken into account when selecting the cows. The genotyped German Brown bulls came from artificial insemination (AI) stations and these AI-bulls also sired daughters in these 258 herds. The animals were genotyped with SNP panels of medium density but different SNP content. All animals were imputed to 50 K using the software BEAGLE 5.4 [32]. The map file with the most SNPs was selected as a reference and SNPs were aligned to the ARS-UCD1.2 reference genome [33]. Consequently, there were no missing genotypes. 

Corresponding pedigree data were obtained from the official milk recording organization of Bavaria (Landeskuratorium der Erzeugerringe für tierische Veredelung in Bayern e.V., LKV, München, Germany) and contained all German Brown animals since the beginning of electronic data recording. The data set included birth and calving date, and where already available, the culling date of the cow was included. At the time of data analysis, there were 1423 cows that had already left the herd.

Animals had on average a breed proportion of US Brown Swiss of 73.82 ± 10.43% with a range from 19 to 98%. All animals were grouped into 5 classes according to their breed proportion of US Brown Swiss: BS <60%, BS 60–69%, BS 70–79%, BS 80–89%, BS 90–99%, with 3.0%, 9.9%, 17.6%, 41.9% and 27.6% of all animals, respectively. 

Cows were also grouped according to survival to a particular lactation as recently described [6], with Surv1, Surv3, Surv5, Surv7 and Surv9 as 0/1 traits and defined as the survival to the second (*n* = 2238), fourth (*n* = 1768), sixth (*n* = 1444), eighth (*n* = 1427) or tenth (*n* = 1426) calving, respectively. Cows were defined as Surv1 (Yes), Surv3 (Yes), Surv5 (Yes), Surv7 (Yes) or Surv9 (Yes) when they survived to the second, fourth, sixth, eighth or tenth calving, respectively. All cows leaving before the second, fourth, sixth, eighth or tenth calving were defined as Surv1 (No), Surv3 (No), Surv5 (No), Surv7 (No) or Surv9 (No), respectively. All other cows were treated as missing. A further grouping distinguished the cows by their last lactation number when milk recordings were registered. This grouping classified cows that did not survive to the second lactation and those that only had milk up to the second, fourth, sixth to eighth, ninth to twelfth and thirteenth to seventeenth lactation (Lact1, Lact2, Lact4, Lact6–Lact8, Lact9–Lact12, and Lact13–Lact17). 

### 2.2. Detection of Runs of Homozygosity

For the identification of runs of homozygosity (ROH), we used the sliding window approach in PLINK (www.cog-genomics.org/plink/1.9/ (accessed on 19 February 2024)), version 1.9, Complete Genomics, Mountain View, CA, USA [34]. As suggested by Meyermans, et al. [35] for ROH-analyses using medium density SNPs, we did not prune by LD, minor allele frequency (MAF) or Hardy–Weinberg equilibrium (HWE). In addition, we tested different parameter sets to indicate the impact on the estimates of F_ROH_ when constraints were applied to MAF and HWE for the genotype data. This would have led to an underestimation of homozygosity, which would imply a 0.014 reduced F_ROH_ in the population under study (Supplementary Material Table S7). Therefore, the final set consisted of 49,693 SNPs for further analysis. The distribution of the SNPs by autosomes is given in Supplementary Material Table S8.

To minimize the number of detected ROH that are only identical by state but not identical by descent, the minimum number of SNPs was calculated as suggested by Lencz, et al. [36]:

$$l=\frac{\log_{e}\frac{\alpha }{n_{s}n_{i}}}{\log_{e}\left(1-het\right)}$$

where α is the percentage of false positive ROH, which was set to 0.05; *n_s_* and *n_i_* are the number of SNPs and the number of animals, respectively; and *het* is the average SNP heterozygosity. Based on the average SNP-density (d) of 49.86 kb per SNP, the minimum length was calculated by multiplying the SNP density with the minimum number of SNPs and set at 3191 kb. Reducing the minimum number of SNPs to 15 and/or setting the minimum length to 1 Mb, while keeping the other parameters constant, as defined in previous papers (Supplementary Material Table S9), would have resulted in higher estimates for F_ROH_ as shown in Supplementary Material Table S7. This could represent overestimation by randomly determined ROH based on medium density SNP data. The maximum gap of 500 was chosen as it was recommended to minimize the gap length [35]. We also tested the effect of a gap length of 1000 Mb, which led to minimally higher results for F_ROH_ (Supplementary Material Table S7). Since window sizes of 20, 35 and 50 SNPs tend to underestimate true inbreeding with respect to higher generations according to Forutan, et al. [37], we chose a window size of 15 SNPs. Nevertheless, the effects on F_ROH_ were small, ranging from 0.125 to 0.116 for window sizes of 10 and 30 SNPs (Supplementary Material Table S7). The final PLINK command reads: -homozyg-kb 3191 --homozyg-snp 64 --homozyg-density 100 --homozyg-gap 500 --homozyg-window-snp 15 -homozyg-window-het 1 --homozyg-window-missing 0-.

The identified ROH were grouped in 5 length classes, which were ≤4 Mb, >4–8 Mb, >8–16 Mb, >16–32 Mb and >32 Mb. 

### 2.3. Inbreeding and Effective Population Size

Based on the pedigree data, classical inbreeding (F_PED_) and ancestral inbreeding coefficients according to Kalinowski, et al. [38] (F_a_Kal_, F_New_), Ballou [39] (F_a_Bal_,) and Baumung, et al. [40] (Ahc) were calculated as described previously [5] using the software packages PEDIG, version 5, [41] and GRAIN, version 2.2 [40].

The inbreeding coefficient F_ROH_ was defined as the proportion of the sum of the length of ROH of all autosomal ROH of an individuum (∑L_ROH_) with a minimum length of 3191 Kb and covered by 64 SNPs and the total length of the autosomes (L_AUTO_) covered by SNPs, which was 2,477,795,166 bp for the analyzed data [42].

$$F_{\mathrm{ROH}}=\frac{\sum L_{\mathrm{ROH}}}{L_{\mathrm{AUTO}}}$$

In addition, F_ROH_ was calculated on the basis of different minimum lengths of 4 Mb, (F_ROH>4_), 8 Mb (F_ROH>8_), 16 Mb (F_ROH>16_) and 32 Mb (F_ROH>32_). These F_ROH_, restricted in their minimum length, capture inbreeding up to approximately 12.5, 6.25, 3.125 and 1.5625 generations. 

The excess of homozygosity F_IS_ was calculated as:

$$F_{\mathrm{IS}}=\frac{O_{i}-E_{i}}{n_{SNP,i\;}-E_{i}}$$

where *O_i_* is the number of observed homozygous SNPs of individual *i*, *E_i_* is the expected homozygous SNPs of individual *I*, and *n_SNP_* is the number of all genotyped autosomal SNPs of individual *i* [43].

The effective population size N_e_ was defined based on linkage disequilibrium and the intergenetic marker distance expressed as the squared correlation between two SNPs (r^2^) and the recombination rate c [44]. Thus, r^2^ was calculated using PLINK (www.cog-genomics.org/plink/1.9/), version 1.9, Complete Genomics, Mountain View, CA, USA [34]. We grouped the r^2^-values for SNP pairs with distances of 1 kb to 33.3 Mb in distance bins of 0.1 Mb. The mean r^2^-value was calculated for each bin and the effective population size was estimated as $N_{e}=\frac{1-r^{2}}{4cr^{2}}$, with c = recombination rate in Morgan units [44]. We assumed that 100 Mb = 1 Morgan. The number of ancestral generations *t* was calculated as $t=\frac{1}{2c}$ and rounded to the nearest integer. The values were averaged if there was more than one value of *N_e_* per integer. The rate of inbreeding per generation was accordingly calculated as $\Delta F=\frac{1}{2N_{e}}$. In addition, we calculated ΔF and *N_e_* using F_ROH_, F_ROH>4_, F_ROH>8_, F_ROH>16_ and F_ROH>32_ adjusted to the numbers of known equivalent generations underlying the respective F_ROH_ [45] as:

$$\Delta F_{ROH-i}=1-\sqrt[{GE-ROH_{i}-1}]{(1-F_{ROH-i}})\;and\;N_{e-ROH-i}=1/2\Delta F_{ROH-i}$$

with F*_ROH-i_* = F_ROH_, F_ROH>4_, F_ROH>8_, F_ROH>16_ and F_ROH>32_. The number of known equivalent generations (GE-ROH_i_) was derived from the minimum length of ROH, which correspond to 15.6691, 12.5, 6.25, 3.125 and 1.5625 generations for F_ROH_, F_ROH>4_, F_ROH>8_, F_ROH>16_ and F_ROH>32_, respectively. 

We also used the linear regression coefficient of the natural logarithm of (1 − F_ROH_) on time in years to calculate the rate of inbreeding per year as ΔF_yr_ = 1 − exp(b), with b = linear regression coefficient, and the effective population size N_e-reg_ [46] with its 95% confidence intervals using the generation interval in years (L = 6.5 years) and the standard error of the linear regression coefficient (SE_b_) as:

$$N_{e-\mathrm{reg}}=1/(2\times L\times \Delta F_{yr})\;\mathrm{and}\;\mathrm{its}\;95\%\;\mathrm{CI}\;\mathrm{as}\;1/(2\times L\times ({\Delta F}_{yr}\pm 1.96\times SE_{b})).$$

### 2.4. Statistical Analysis

Statistical analyses were performed using SAS, version 9.4 (Statistical Analysis System, Cary, NC, USA, 2023). We used the SAS procedures means, freq, glm, and ttest with the option bootstrap and the procedure corr with the option α. The inbreeding coefficients were tested a priori for their distribution and all were normally distributed. Least squares means for the descriptive ROH parameters and inbreeding coefficients by US Brown Swiss classes were calculated using the following linear model:

*y*_ij_ = µ + BS_i_ + e_ij_ (1)

where y_ij_ = dependent variable with the mean number of ROH, the average ROH length, the total length of ROH, and F_IS_, F_ROH_, F_ROH>4_, F_ROH>8_, F_ROH>16_ and F_ROH>32_. BS_i_ = is the fixed effect of the ith US Brown Swiss class for i = 1 (BS <60%, *n* = 70), 2 (BS 60–69%, *n* = 228), 3 (BS 70–79%, *n* = 407), 4 (BS 80–89%, *n* = 967), 5 (BS 90–99%, *n* = 636) and e_ij_ = random error term. For the analysis by lactation numbers that the cows reached, the fixed effect of Lact_i_ with six classes (Lact1, *n* = 434; Lact2, *n* = 494; Lact4, *n* = 331; Lact6–Lact8, *n* = 64; Lact9–Lact12, *n* = 196 and Lact13–Lact17, *n* = 69) was parameterized in a linear model as follows:

*y*_ij_ = µ + Lact_i_ + e_ij_

For the analysis by survival class, only cows with a reported culling date were included in the survival traits, and in the model, only the fixed effect of survival to the second calving (Surv1), fourth calving (Surv3), sixth calving (Surv5), eighth calving (Surv7) or tenth calving (Surv9) as 0/1-trait with j = 1 (No), 2 (Yes) was considered.

To account for the combined effect of the breed proportion of US Brown Swiss and survival to the second, fourth, sixth, eighth and tenth calving, we also tested a linear model including the two-way interaction of BS x Surv_ij_ as a fixed effect. In addition, we used the procedure ttest with the option bootstrap and the procedure corr of SAS, version 9.1.4. 

### 2.5. ROH Islands, Consensus ROH and Gene Ontology Enrichment

We evaluated the frequency of animals with SNPs within ROH. The regions exceeding the 99th percentile of the homozygosity distribution were defined as ROH islands. We also examined ROH islands, separately for the five US Brown Swiss classes (BS <60%, BS 60–69%, BS 70–79%, BS 80–89%, BS 90–99%) and survival classes (Surv1 = Yes/No, Surv 3 = Yes/No, Surv5 = Yes/No, Surv7 = Yes/No and Surv9 = Yes/No). We also extended the ROH island definition to the 95th percentile to capture a larger proportion of potential ROH islands. Furthermore, we screened for consensus ROH that were shared by at least 30%, 40% and 50% of the animals. 

Annotated genes located in ROH islands and consensus ROH were retrieved from the Ensemble genome assembly, release 110 [47] and were further analyzed using PANTHER, version 17.0 (Protein Analysis Through Evolutionary Relationships), Division of Bioinformatics, Department of Preventive Medicine, Keck School of Medicine of USC, University of Southern California, Los Angeles, CA, USA [48] to identify their molecular functions and the biological processes. In addition, previously described QTL within the detected ROH islands were screened using the Animal QTLdb [49] (https://www.animalgenome.org/cgi-bin/QTLdb/BT/index) accessed on 2 March 2024.

## 3. Results

### 3.1. Descriptive Statistics for ROH and Inbreeding for All Animals

The average number of ROH per animal was 35.996, whereby a ROH was on average 8.323 Mb long (Table 1).

**Figure 1:**
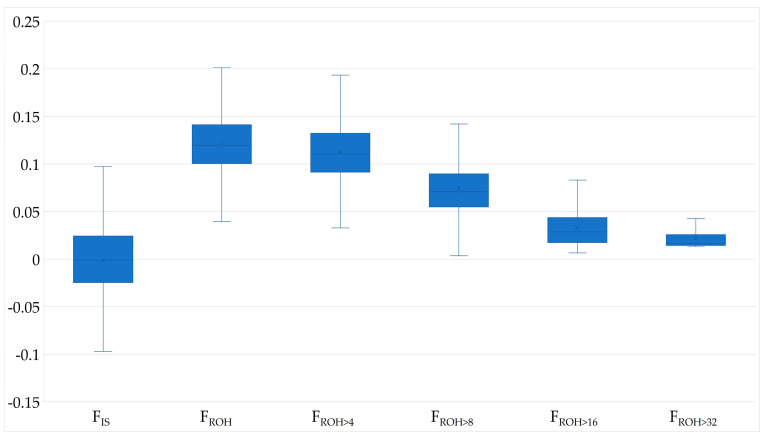
Boxplots of genome based inbreeding coefficients with F_IS_, F_ROH_, F_ROH>4_, F_ROH>8_, F_ROH>16_ and F_ROH>32_ for German Browns.

The mean F_ROH_ was 0.122 ± 0.032 and the mean F_IS_ was −0.001, with 1207 animals (51.1%) having a F_IS_-Value below 0. Most ROH belonged to ROH length class 4–8 Mb (47%) and only 9% of the ROH were longer than 16 Mb. F_ROH>16_ was 0.031 and corresponds to about a quarter of F_ROH_. However, ROH longer than 8 Mb were not detected for all cows; thus F_ROH>16_ and F_ROH>32_ were absent for 4.7% and 71.45% of the animals, respectively. The mean F_PED_ was lower than F_ROH_, but both increased in parallel with the birth years (Table 2, Figure 1, Supplementary Material Figure S1). Ancestral inbreeding ranged from 0.014 to 0.123, with F_New_ being twice as high as F_a_Kal_. 

F_PED_ and F_ROH_ were moderately correlated with 0.572 and Pearson correlation coefficients between F_PED_ and F_ROH>4_, F_ROH>8_, F_ROH>16_ and F_ROH>32_ decreased with increasing minimum ROH length (Table 3). The correlation coefficient between F_PED_ and F_IS_ was 0.571, while a high correlation of 0.925 was observed between F_ROH_ and F_IS_, which decreased to 0.469 between F_ROH>32_ and F_IS_. F_a_Bal_ and F_IS_ had a higher correlation coefficient of 0.467 than F_a_Bal_ and F_ROH_ (0.395) and the lowest correlation was observed between F_a_Bal_ and F_ROH>32_ (0.084). F_a_Kal_ and F_New_ showed comparable correlation coefficients with F_ROH_, which were 0.535 and 0.533, respectively, but with increasing minimal ROH length, correlations of F_a_Kal_ and F_New_ with F_ROH>32_ decreased to 0.265 and 0.396. Correlations with the proportion of US Brown Swiss genes reached estimates from 0.099 to 0.153 for pedigree-based inbreeding coefficients, while correlations with F_ROH_ were at 0.070 and not significantly different from zero with F_ROH>16_ and F_ROH>32_. 

**Figure 2:**
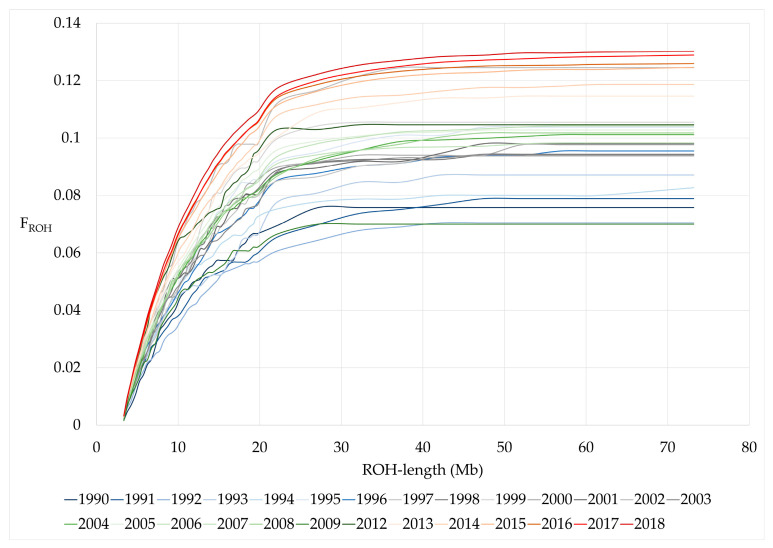
Cumulative F_ROH_ by birth years.

The cumulative distribution of F_ROH_ by ROH lengths showed a steadily increasing steeper increase up to a length of 20 Mb in animals from younger birth years compared to animals born in earlier years (Figure 2).

### 3.2. ROH and Inbreeding by US Brown Swiss Classes

Least squares means by US Brown Swiss classes significantly increased for the average number of ROH from BS <60% to BS 90–99%, with significant differences between BS <60% and BS 60–69%, BS 80–89% and BS 90–99%. The combined ROH length was significantly shorter for BS <60% compared to BS 60–69%, BS 80–89% and BS 90–99% (Table 4).

**Figure 3:**
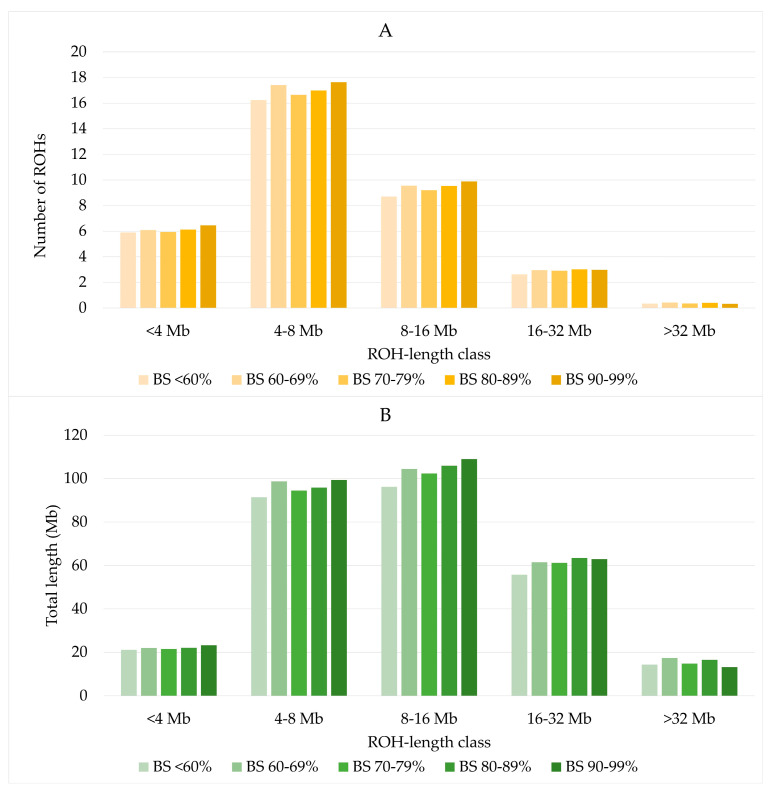
Number of ROH (**A**) and total length of ROH (**B**) by US Brown Swiss classes.

The distribution of ROH by length classes was similar across all US Brown Swiss classes (Figure 3A). ROH with a length of 8–16 Mb covered a slightly larger part of the genome than ROH of length classes of 4–8 Mb (Figure 3B). The lowest number and lowest combined ROH lengths were found for ROH longer than 32 Mb.

**Figure 4:**
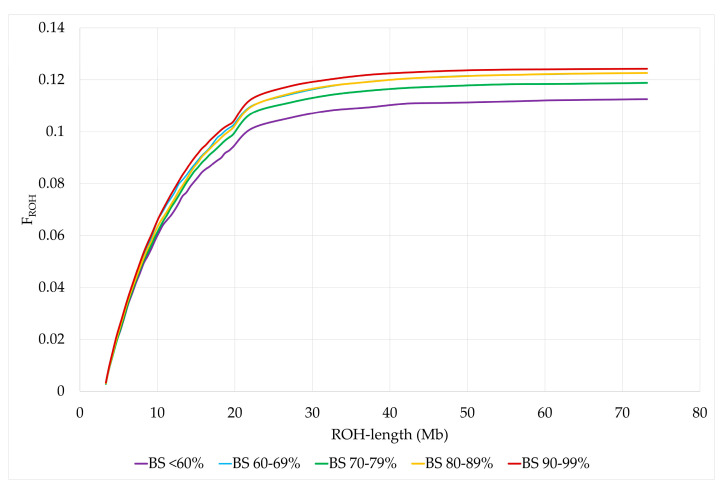
Cumulative distribution of F_ROH_ by US Brown Swiss classes.

Least squares means (LSM) for F_ROH_ of animals in BS <60% were significantly lower than those of animals with BS 60–69%, BS 80–89% and BS 90–99%. In addition, LSM of F_ROH_ were significantly higher for BS 90–99% than for BS 70–79%. The same distribution was observed for F_ROH>4_, while the differences for F_ROH>8_ were only significant between BS <60% and BS 80–89% and no significant differences were found between US Brown Swiss classes for F_ROH>16_ and F_ROH>32_ (Figure 4 and Table 5). The standard errors and 95% confidence intervals were lowest for BS 80–89%, since the number of animals in this BS class was the largest, while the opposite was true for BS <60% (Supplementary Material Table S10). In addition, we used bootstrapping to resample the standard errors and 95% bootstrap bias-corrected confidence intervals for the sample mean estimates of the differences between each two US Brown Swiss classes for the ROH parameters (Supplementary Material Table S11). The bootstrap standard errors and 95% bootstrap bias-corrected confidence intervals for the estimated differences were the largest for BS <60% and the smallest for BS 80–89%. Despite the smaller sample size for BS <60%, differences to BS 60–69%, BS 80–89% and BS 90–99% were significantly different for the average number of ROH, F_IS_, F_ROH_ and F_ROH>4_. Furthermore, we used Cronbach’s α coefficient as a measure for the reliability of the consistency for genomic measures of inbreeding (F_IS_, F_ROH_, F_ROH>4_, F_ROH>8_, F_ROH>16_, and F_ROH>32_) per BS class. For BS <60%, BS 60–69%, BS 70–79%, BS80–89 and BS 90–99%, the Cronbach’s α coefficient was 0.946, 0.943, 0.940, 0.943 and 0.933, respectively.

### 3.3. ROH and Genomic Inbreeding by Survival Classes

**Figure 5:**
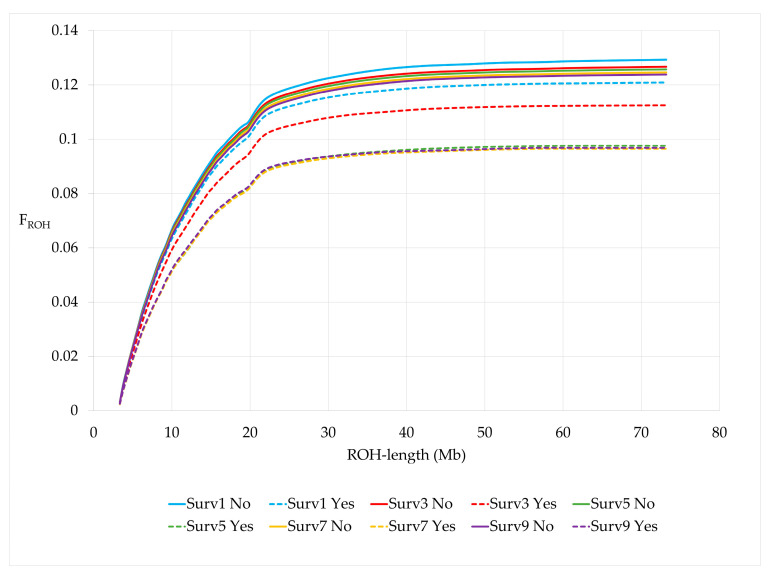
Cumulative F_ROH_ by survival traits.

When cows were grouped by survival to the 2, 4, 6, 8 and 10th lactation, cows surviving the respective lactation number (Surv, Yes) had a significantly lower number of ROH and a shorter average ROH length compared to non-survivors (Surv, No) for all survival traits (Table 6). F_ROH_ was higher for non-survivors and these cows showed a steeper slope than survivors for all survival traits (Figure 5). In addition, F_ROH_ significantly decreased in cows surviving three or more lactations compared to cows surviving just the first lactation. The same was found for the other F_ROH_ with a larger minimum length of homozygous genomic segments. Differences in genomic inbreeding for all different F_ROH_ definitions were largest for the trait surv5 (Table 6, Supplementary Material Figure S2).

When animals were grouped by survival traits and US Brown Swiss classes, survivors had lower genomic inbreeding in all US Brown Swiss classes, which were significant for F_ROH_ and F_ROH>4_ with the exception of surv1 in BS <60% (Supplementary Material Table S12). In addition, cows that survived lactation 3, 5, 7 and 9 had significantly lower F_ROH_ in BS <60% compared to cows in BS 90–99%, whereas the corresponding differences between the other US Brown Swiss classes were not significant for non-surviving cows (Supplementary Material Table S12). This was also the case for F_ROH>4_ and F_ROH>8_. 

The analysis of the lactation numbers the cows reached resulted in similar estimates as for the survival traits (Supplementary Material Table S13). Genomic inbreeding coefficients were lowest for cows that completed 13–17 lactations. The estimates for F_ROH_, F_ROH>4_, F_ROH>8_, F_ROH>16_ and F_ROH>32_ were at 0.0917, 0.0844, 0.0536, 0.0196 and 0.0018, respectively, and significantly different (*p*-value < 0.001) from the corresponding estimates of cows that did survive to the second lactation. 

### 3.4. Consensus ROH and ROH Islands

Consensus ROH observed in 30% of the animals were located on BTA 4, 5, 6, 11, 16 and 19, and contained 16 different ROH (Supplementary Material Table S14a). For 40% of the animals, eight consensus ROH were found on BTA 5, 6, and 16 (Supplementary Material Table S14b). Only one consensus ROH on BTA6 was retrieved for 50% of all animals (Supplementary Material Table S14c). The longest ROH segments for the 30% consensus ROH were located on BTA 6 between 73 and 95 Mb, on BTA 5 between 71 and 85 Mb, and on BTA 16 between 21 and 31 Mb containing 381, 260 and 182 SNPs, respectively. The region on BTA 6 from 85 to 88 Mb containing *ADAMTS3* was shared by at least 50% of the animals (Supplementary Material Table S14c). 

ROH islands above the 99th percentile threshold were found on BTA 5, 6 and 16 (Table 7, Supplementary Material Table S15). Analysis of ROH islands separately for US Brown Swiss classes revealed similar regions. Compared to animals with a higher breed proportion of US Brown Swiss, the ROH island on BTA 16 was very small in animals of BS <60%, with only five SNPs and two annotated genes (Table 7). Animals of BS 60–69% also had a ROH island on BTA 5 between 12 and 24 Mb. Analysis of cows by survival traits showed that the ROH islands on BTA 6 between 48 and 50 Mb and on BTA 16 between 21 and 29 Mb were no longer present in Surv5, Surv7 and Surv9 (Table 7). 

If ROH islands are defined as above the 95th percentile threshold, the number of ROH islands increased from 4 to 18 and the size of the ROH islands of the 99th percentile threshold increased when compared to those of the 99th percentile threshold. However, 10/18 of the ROH islands of the 95th percentile threshold were not present in the cows with BS <60%, while 7/18 of the ROH islands of the 95th percentile threshold were found in all US Brown Swiss classes (Supplementary Material Table S16a–c). The number of ROH islands by US Brown Swiss classes amounted to 43. We found 7/43 ROH islands specific for BS <60% on BTA 2, 3, 6, 14 and 23; while for BS 60–69% on BTA 4, for BS 80–89% on BTA 21, and for BS 90–99% on BTA6, only one ROH island each was specific. Four ROH islands only occurred at BS <80% and three ROH islands only at BS >79%.

Considering survival traits, one ROH island was exclusively discovered on BTA 7 for Surv9 and on BTA 11 for Surv1 (Supplementary Material Table S17a,b).

The distribution of ROH islands above the 99th percentile threshold for cows by completed lactation number Lact2, Lact4 and Lact6–Lact8 and Lact9–Lact12 was similar to that for cows by survival traits (Supplementary Material Table S18). The cows in the Lact13–Lact17 group showed four ROH islands on BTA 5, 6 and 16, whereas cows of the Lact9–Lact12 group showed the same ROH islands as the cows that survived lactations 5, 7 and 9.

Results of the PANTHER Gene ontology enrichment analyses of the ROH islands are presented in Supplementary Material Table S19. The most common molecular functions and biological processes for the ROH islands were ‘binding’, ‘catalytic activity’, ‘cellular process’, ‘biological regulation’, and ‘metabolic process’, respectively. We tested for enrichment of the ROH islands and found genes in three ROH islands on BTA 5 (74–78 Mb), BTA 6 (73–91 Mb) and BTA16 (21–29 Mb) that were significantly overrepresented. These genes were proposed to have effects on milk production, casein types, mastitis and disease resistance, longevity and meat quality [18,19,29,30,31]. 

### 3.5. Effective Population Size 

**Figure 6:**
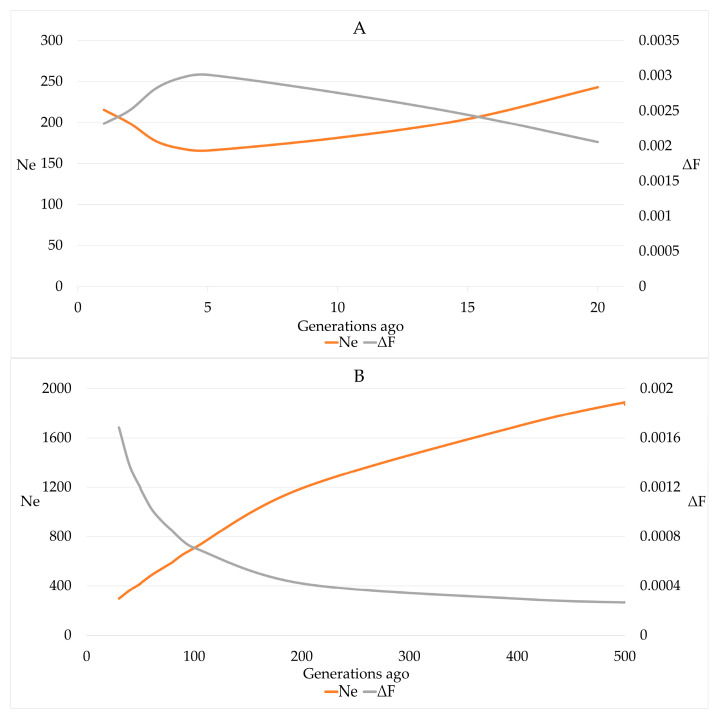
Effective population size (N_e_) and increase in inbreeding (ΔF) in German Brown cows for 20 generations (**A**) and 20–500 (**B**) generations ago based on linkage disequilibrium between consecutive SNPs.

Estimates for r^2^ ranged from 0.002 to 0.774 for SNP distances of 49.7 Mb and 1.5 kb, respectively. The estimates of N_e_ that was calculated based on r^2^ declined from 243 for 20 generations ago to 166 for 5 generations ago. Within the last 5 generations, an increase of N_e_ for the most recent generation to 215 was observed (Figure 6). 

The estimates for N_e-ROH_ were 56.870, 48.203, 34.394, 33.991 and 46.985 when based on F_ROH_, F_ROH>4_, F_ROH>8_, F_ROH>16_ and F_ROH>32_, respectively. Using the slope on time of the natural logarithm of (1 − F_ROH_), we obtained estimates of 0.00119 ± 0.00032 for ΔF_yr_ and 76.373 for N_e-reg_ with a 95% CI of 49.857 to 163.140. The average length of ROH segments remained constant over the birth years, whereas the number of ROH segments significantly (*p*-value < 0.0001) increased by 0.3541 ± 0.0664 by year and 2.301 by generation.

## 4. Discussion

In this study, we aimed to characterize the patterns of ROH in German Brown cattle based on 50 K SNP data with special emphasis on the influence of the breed proportion of US Brown Swiss and survival rates for the 1st to 9th lactation in order to find genomic regions associated with survivability to high lactation numbers and whether the introgression of US Brown Swiss did effect survival rates.

The effective population size calculated in this study based on LD showed similar patterns to Italian Holsteins, where an increase from generation 5 to the youngest generation was reported [10], but the effective population size was lower for Italian Holsteins with estimates of 96 and 120 for generations 6–9 and 120 in the youngest generation, respectively. A lower N_e_ was also found for Italian Brown bulls, namely 237.6 and 90.7 for generation 50 and 5, respectively [50] and for Canadian Holsteins and Jerseys with N_e_ estimates of 58 and 120 for generation 5, respectively [51]. In agreement with results for Italian Modeneses [13] and US Holsteins and Jerseys [51], N_e_ estimates using regression on time resulted in lower values. Even smaller N_e_ estimates were obtained from the realized rate of inbreeding. Nevertheless, the N_e_ estimates exceeded the critical value of 50, at which long term negative effects due to inbreeding depression may be expected [5,10,13,51].

When comparing different studies on ROH analyses, different ROH definitions complicated the interpretation. In previous studies analyzing ROH in Brown populations, the minimum number of SNPs ranged from 15–60, with a minimum length between 1–2 Mb, allowing either one or no heterozygous or 0–2 missing SNPs [17,18,19,29,31,49,52]. In addition, in most studies, a pruning for MAF was performed. As this could lead to an underestimation of genomic inbreeding, this was not considered in this study, possibly leading to higher inbreeding estimates [35,37,52]. The minimum number of SNPs and the minimum length in this study were calculated based on the number of SNPs, the number of animals, and the average heterozygosity, and are thus higher than most of the studies, being most consistent with those in the study on US Brown Swiss [52]. In agreement with previous studies [35,37,52], it may be expected that in our data, when applying a lower minimum number of SNPs, a lower minimum length of ROH, a larger gap size between consecutive ROH, a smaller window size and without pruning for MAF, more ROH will be detected, resulting in higher genomic inbreeding coefficients (Supplementary Material Table S7). 

In German Browns, almost half of the ROH were between 4 Mb and 8 Mb long, which are assumed to correspond to 12.5 and 6.25 ancestral generations ago, assuming 1 cM = 1 Mb. In Original and modern Brown populations, the most abundant ROH class was 4–8 Mb [18], and in Swiss Brown populations 5–10 Mb [29]. In Italian Brown Swiss [19,50] and US Brown Swiss [52], a decreasing number of ROH was found with increasing ROH length class, so that most ROH were 1–2 Mb and 2–4 Mb respectively. Since the minimum ROH length in the present study was set to 3.191 Mb, ROH of 1 to 3 Mb were not considered in this study. Using the ROH settings of the US study [52] but without MAF pruning, we observed in our data an increase of ROH segments with 3–4 Mb and a shift of the frequency from 17.04% to 26.90% for the ROH class < 4 Mb across all animals. The number of ROH < 4 Mb by US Brown Swiss classes increased from 5.9–6.5 to 10.6–11.5 in the present study.

Nevertheless, the average length of a ROH of 8.323 Mb is comparable to the average length for Italian Browns of 8.54 Mb [18]. In US Brown Swiss, the average ROH length was slightly lower at 7.54 Mb, with a higher average number of ROH at 47.69 [52]. Applying the definitions for ROH like in the US study [52] but without MAF pruning, the average ROH length decreased to 7.657 Mb and the average number of ROH increased to 40.709 in our data. Therefore, the reason for this difference may be mainly due to the definition of ROH and, to a lesser extent, that more detected ROH in US Brown Swiss belong to shorter ROH length classes, accounting for inbreeding in former times. Therefore, short ROH < 3.191 Mb, which were not retrieved in our study, led to a larger number of ROH of smaller sizes in US Brown Swiss. Nevertheless, studies comparing ROH of Original Browns to modern brown breeds reported a lower average ROH length [18] and a lower average number of ROH [29] for the Original Browns. Although we did not study animals of different Brown cattle subpopulations, the evaluation according to different US Brown Swiss classes showed a similar trend for the distribution of ROH by length classes. A higher average number of ROH per animal was found for animals in BS 90–99% compared to animals of <60% and 70–89% breed proportion of US Brown Swiss. However, ROH islands of the 99th percentile threshold were not specific for US Brown Swiss classes. All four ROH islands of the 99th percentile threshold found for the entire sample were also present in each of the US Brown Swiss classes. Nevertheless, a greater differentiation of the genomic architecture between US Brown Swiss classes was found when searching for ROH islands of the 95th percentile threshold. When comparing the 18 ROH islands of the 95th percentile threshold identified in the entire sample, 7/18 ROH islands of the 95th percentile threshold were equally distributed across all US Brown Swiss classes and 5/18 across four US Brown Swiss classes. Within US Brown Swiss classes, a total number of 43 ROH islands was identified. There were 7/43 ROH islands common to all US Brown Swiss classes, 11/43 ROH islands common to BS <70% and 9/43 ROH islands frequent in BS >69%. In summary, the distribution of the ROH islands of the 99th percentile threshold indicates changes in the size of the ROH islands between US Brown Swiss classes, but not to such an extent that animals in BS 90–99% can be considered a distinct subpopulation. Even 67% of the 18 ROH islands of the 95th percentile threshold, as shown for the entire sample, are shared by all five or at least four US Brown Swiss classes. Nevertheless, ROH analyses within US Brown Swiss classes suggested that animals of the different US Brown Swiss classes differ in 10/43 ROH islands of the 95th percentile threshold and there may be trait loci, which may be specific for traits of the animals in these respective US Brown Swiss classes (Supplementary Material Table S20). Most distinct ROH islands of the 95th percentile threshold were found in BS <60% (7/10) and only 1/10 in BS 90–99%. 

The proportion of animals sired by US Brown Swiss bulls amounted to 4.47% in the German Brown birth cohorts from 1990–2018. German Brown sires are therefore mostly crosses with US Brown Swiss bulls. The 75% and 95% confidence intervals for the proportion of US Brown Swiss genes in the sires for the 1990–2018 birth cohorts of the German Brown population were 69–88% and 58–97%, respectively. This may explain why the ROH islands of the 95th percentile threshold were found in all five US Brown Swiss classes and 12/18 of the ROH islands of the 95th percentile threshold were found in at least four US Brown Swiss classes. 

The average F_ROH_ in the present study was higher than what has been reported for Original Brown, Braunvieh (Brown cattle with incrossings of US Brown Swiss bulls) and US Brown Swiss sires in Switzerland with F_ROH_ of 0.029, 0.074 and 0.091, respectively [29]. Higher values for F_ROH_ of 0.15 were found for US Brown Swiss in the US [52]. Using the same settings for defining ROH as in the latter two studies, the same tendency for F_ROH_ was confirmed (Supplementary Material Table S7). 

The increasing trend in F_ROH_ and pedigree based inbreeding coefficients across birth years for the 2364 animals in this study is consistent with our recent study in German Browns based on pedigree data, in which the inbreeding coefficients increased from 0.013 to 0.036 between 1990 and 2014 [5]. The average values for lifetime traits and pedigree-based inbreeding coefficients from the present data set also agree well with the entire German Brown population (Supplementary Material Table S6). The animals in this study therefore represent a random sample from the entire population. 

Based on the pedigree data, the relationship of F_New_ and F_PED_ shows that more than two thirds of the inbreeding comes from alleles that are IBD for the first time and the high correlations between F_New_ and F_PED_ underline the large influence of inbreeding from more recent generations. The correlation between F_New_ and F_ROH_ was rather similar to that of F_ROH>4_ and F_ROH>8_, while the correlations between F_New_ and F_ROH>16_ or F_ROH>32_ were considerably smaller. Considering the correlation between F_ROH>8_ and F_ROH_ with an estimate of 0.936, which corresponds to the correlation coefficient between F_New_ and F_PED_, it can be assumed that a large portion of new inbreeding is covered by ROH segments between 8–16 Mb, i.e., the ROH length class with the highest total length. This relates to 3.125 to 6.25 generations. Underlying a generation interval of 7 years, this corresponds to a period of 22 to 44 years and falls into the time after the beginning of the introgression of US Brown Swiss in 1966, i.e., 24 to 52 years ago in relation to the birth years 1990–2018 in this study. Along this line, correlation coefficients between the different F_ROH_ and proportion of US Brown Swiss genes decreased with longer ROH segments. Therefore, it is likely that ROH < 6.25 Mb originated from Original Brown ancestors prior to the introduction of US Brown Swiss. These results support the outcomes of our previous study: that the breeding scheme during that time increased the level of inbreeding due to the unbalanced use of a few top US Brown Swiss sires and contributed to the current level of inbreeding [5]. Nevertheless, systematic mating of closely related individuals cannot have taken place on a large scale as the F_IS_ value is negative and close to zero. 

The tendency toward higher F_ROH_ with increasing breed proportion of US Brown Swiss supports the impact of US Brown Swiss on inbreeding. However, the lack of differences of F_ROH>16_ and F_ROH>32_ between the US Brown Swiss classes and their correlations close to zero also indicate that in more recent generations the effect of using US Brown Swiss genetics has lost its impact on inbreeding levels in the German Brown population. The fact that not all animals showed ROH of more than 16 Mb could also explain the lack of significant differences. In studies comparing Original Browns with Brown Swiss, higher F_ROH_ values were also reported for the modern Brown Swiss populations [28,29].

Testing for significant differences is influenced by samples sizes (*n*) since sample size is connected with the size of standard errors ($SE=SD/\sqrt{n}$, with SD = standard deviation). This may have had an influence on the non-significant *p*-values when comparing average numbers of ROH, F_IS_, F_ROH_ and F_ROH>4_ between BS <60%, with a small sample size of 70, and BS 70–79%. A larger sample size for BS <60% at >200 would have resulted in significant *p*-values. Rather uneven sample sizes are also common in previous reports on ROH [17,18,19,20,28,29,30]. 

It has already been shown that the proportion of US Brown Swiss genes is associated with longevity [3], and that longevity is negatively associated with inbreeding [6]. Considering survival to the following calving, cows that left the herd earlier had significantly higher F_ROH_ values compared to those surviving until the following calving. This means that cows that are more inbred have a higher risk of leaving the herd. Cows that survived to at least the 5th to the 9th lactation exhibited the lowest levels of inbreeding. This relation is consistent with the results based on pedigree data [6]. When accounting for the effect of the interaction of US Brown Swiss classes with survival, the differences between the survivors and not survivors were largest for F_ROH_ and BS <60% but decreasing in the BS classes with more US Brown Swiss genetics (Supplementary Material Table S13). We may propose that inbreeding may be more detrimental for survival to high lactation numbers in German Brown cows when the proportion of US Brown Swiss is decreasing, particularly <60%. Recent inbreeding should go to zero independent of the US Brown Swiss proportion in order to increase the possibility of long survival times of the cows. Genomic inbreeding was lowest for cows with BS <60% having survived 5, 7 and 9 lactations and went to zero for F_ROH>32_.

The region of strongest selection was detected on BTA 6 between 73 and 91 Mb, which was the longest ROH island and the longest consensus ROH shared by approximately 30% of the animals, with the region between 85 and 88 Mb shared by approximately 50% of the animals. In this genomic region, the casein genes are located, which are central for dairy cattle breeding. The casein genes *CSN1S1*, *CSN2*, *CSN1S2* and *CSN3* have been associated with milk production [23] and milk protein percentage [53] in European Brown breeds and other dairy cattle populations [23,53,54]. The *CSN3* gene was within the ROH shared by 50% of the animals and encodes kappa casein, which is particularly important for the coagulation properties required for cheese production [55]. Also, a QTL for milking speed was identified in this region [24]. 

Furthermore, in Brown cattle populations, genome wide association studies have found this region to be associated with mammary gland morphology [23], particularly for udder length and teat diameter [21]. The region between 85 and 88 Mb has also been associated with genes involved in udder health and morphology in different dairy breeds such as *SLC4A4*, *NPFFR2*, GC and *RASSF6* [56,57,58,59] and longevity (*NPFFR2* and *ADAMTS3*) [60,61,62], both important traits for German Brown cattle breeding. In agreement with our study, this region was also most frequently reported in studies analyzing ROH islands or selection signatures in modern Brown cattle populations. A selection signature in German Browns using the Cross Population Extended Haplotype Homozygosity (XP-EHH) was detected between 84 and 96 Mb [30]. Also, in Italian Brown Swiss, where selection signatures were identified for SNPs with a ROH count per SNP >50%, a signature around 85 Mb was detected [19]. In a study comparing Original Brown and modern Brown breeds in Italy, a ROH island, defined as the top 0.999 SNPs of the percentile distribution, was detected on BTA 6 between 86 and 87 Mb for modern Brown breeds [18]. In US Brown Swiss, BTA 6 was also reported as a ROH island [52]. 

Also, the region on BTA5 between 71 and 86 Mb was discovered in previous studies as a ROH island in US Brown Swiss and Italian Brown Swiss [18,29,52]. This region harbors QTLs [63,64] and genes primarily associated with milk production such as *NCF4*, *RAC2* and *CSF2RB* [65,66], and is therefore also a region of particular interest in dairy cows.

The ROH island on BTA 16 in the German Brown overlapped the smaller ROH island found in US Brown Swiss [52], suggesting that it originated from US Brown Swiss. This is also supported by the decline of this ROH island with decreasing breed proportion of US Brown Swiss, where for BS <60% this ROH island comprises only 5 SNPs, whereas for BS 90–99% this ROH island reaches its largest size. Moreover, this region overlaps with QTLs mainly associated with milk production and includes genes related to fertility (*TGFB2*) [67] and udder morphology (*SUSD4*) [68]. In addition, the region between 21 and 31 Mb was reported to harbor a selection signature in US Brown Swiss and Swiss Brown in Switzerland [29]. The region on BTA 5 between 12 and 24 Mb was uniquely herein discovered in BS 60–69%, including e.g., the *TMTC2* gene, which was identified as a candidate gene for udder morphology in Brown cattle populations [23].

Nevertheless, the aim of this study was to assess to which extent Original German Brown genes are still under selection pressure. In studies on Original Browns, the region on BTA 11 around 65 to 71 Mb was found to be under strong selection [29,30,31]. This typical Original Brown signature was also detected in the present study as a consensus ROH shared by 30% of the animals at 64 to 69 Mb containing genes associated with meat quality such as *CAPN14* and *CAPN13* and fertility (*PROKR1*) [18,30]. This may suggest that the Original Brown genes are still fixed to a lower extent within the actual German Brown population. Interestingly, when defining ROH islands as the 95th percentile to account for the relatively small number of animals in low US Brown Swiss classes, the region on BTA 11 was detected as a ROH island with nearly the same length across all US Brown Swiss classes. Thus, the homozygosity originating from the Original German Brown animals has been dissolved in about 70% of the actual German Brown population due to the incrossing of US Brown Swiss in a former time, which is consistent with the development from a dual-purpose cattle breed to a more milk-emphasized cattle breed in terms of the associated traits mentioned above. When comparing the ROH islands of the 95th percentile threshold between US Brown Swiss classes, we found that 11 ROH islands, which were present in BS <70%, disappeared in BS >69%, while 5 new ROH islands developed in BS >79%.

Expanding the ROH-definition to the 95th percentile revealed another ROH island on BTA 2 between 76 and 81 Mb which was only detected in BS <60%. This region harbors genes such as *GYPC* and *CNTNAP5* (Supplementary Material Table S16b–d) that have been associated with growth and carcass traits in Beninese indigenous cattle as well as meat quality in Hanwoo [69,70] and milk fat composition [71], representing traits that are more important in beef and dual-purpose cattle breeds than in pure dairy breeds and are therefore likely to be of Original Brown origin. In addition, this region has been reported as a ROH island in an old Polish dual-purpose cattle breed, the Polish Black-and-White [72].

Two further ROH islands on BTA 22 (12–31 Mb) and 28 (15–23 Mb) were detected at BS <60%, BS 60–69% and BS 70–79%, decreasing in length with increasing US Brown Swiss class and absent in animals of the highest US Brown Swiss classes (Supplementary Material Table S16b–d). In Original Browns, SNPs around 24 Mb were associated with multiple births, but not in Brown Swiss [27]. On the other hand, this ROH island was located near the *MITF* gene, which was associated with a white spotted coat color in Brown Swiss [73]. 

The ROH island on BTA 28 has also been found in one of the oldest Polish cattle breeds, the White-Backed breed [72]. Furthermore, this region has been associated with various traits in dairy cattle. Traits such as kappa casein percentage [74], claw health [75,76], and limb and claw conformation, are of particular interest in German Brown cattle breeding [68,77]. Interestingly, the regions on BTA 2, 22 and 28 have also been associated with longevity [68,78,79]. In Chinese Holsteins, a genome-wide association study on nine longevity traits identified the region around 22.9 Mb on BTA 28, which includes *CTNNA3*, as associated with a productive life span from first calving until the end of the first and second lactation [78]. Thus, increasing breed proportions of US Brown Swiss also increase heterozygosity in a region that is likely to affect longevity. 

Increasing heterozygosity was also observed in animals in higher lactation numbers, as the ROH islands based on the 99th percentile on BTA 6 and 16 were only found in animals surviving the third lactation, but not in cows surviving higher lactations.

The definition based on the 95th percentile revealed a ROH island on BTA 7 between 42 and 45 Mb only for cows surviving lactation 9. Since a SNP in this BTA 7 region at position 43,904,171 was associated with herd life in Chinese Holsteins [78], this may indicate that this region may be important for longevity in German Browns. Interestingly, this ROH island on BTA 7 was detected in animals with BS <69% but not in animals with higher proportions of US Brown Swiss genes, supporting the previously reported negative correlation of breed proportion of US Brown Swiss and longevity [3]. The ROH island on BTA 7 overlaps with previously reported QTLs for milk yield [80] and milking speed in French dairy cattle [81], stature and body conformation in Canadian Holstein bulls [82], lean meat yield [83] and somatic cell scores [84]. 

Thus, further investigations of the regions on BTA 7 and 28 with regard to longevity traits in German Brown cows seem necessary to clarify the importance of these regions for longevity traits and to enable the adaption of breeding programs, to prevent further decline of longevity, as an outstanding trait in German Brown cows.

## 5. Conclusions

The analysis of ROH in German Browns showed the focus of selection on milk production traits, udder health and longevity. The ROH islands were shaped by the immigration of US Brown Swiss bulls, but ROH islands originating from Original German Brown cattle were present in animals with fewer US Brown Swiss genes. We propose that ROH stemming from Original Brown cattle may be located on BTA 2, 11, and 28. In these regions, QTLs and genes were identified to be responsible for traits typically found in dual-purpose cattle breeds and having an impact on functional traits including longevity. An exclusive ROH island on BTA 7 was found to be important for longevity in German Brown cows, but this ROH seems to get lost with increasing proportions of US Brown Swiss genes. Our results show that the breeding program should maintain the genetic diversity to which the Original Brown population contributed and breeding aims should give longevity traits and other traits importance for dual-purpose cattle with sufficient weight in the total merit index for bulls and cows.

## Figures and Tables

**Table 1:** Mean, standard deviation (SD), minimum (Min) and maximum (Max) of the average number of ROH, average ROH length and combined length of ROH in all genotyped animals (*n* = 2364).

ROH Items	Mean	SD	Min	Max
Average number of ROH	35.996	7.498	1	63
Average ROH length (Mb)	8.323	1.181	3.793	15.112
Combined length of ROH (Mb)	301.070	80.500	3.794	755.517

**Table 2:** Means, medians, modes, standard deviations (SD) and confidence intervals (CI) of pedigree and genome based inbreeding coefficients.

Inbreeding Coefficients	Mean	SD	Median	Mode	95% CI	75% CI
F_PED_	0.040	0.020	0.038	0.000	0.010–0.072	0.027–0.049
F_a_Bal_	0.111	0.043	0.116	0.000	0.031–0.174	0.084–0.140
Ahc	0.123	0.050	0.128	0.000	0.032–0.200	0.091–0.158
F_a_Kal_	0.014	0.008	0.013	0.000	0.001–0.027	0.008–0.019
F_New_	0.028	0.014	0.026	0.000	0.001–0.051	0.019–0.034
F_IS_	−0.001	0.044	−0.001	−0.020	−0.070–0.064	−0.025–0.024
F_ROH_	0.122	0.032	0.120	0.090	0.072–0.174	0.101–0.141
F_ROH>4_	0.113	0.032	0.113	0.110	0.065–0.165	0.091–0.132
F_ROH>8_	0.074	0.028	0.071	0.000	0.034–0.122	0.055–0.090
F_ROH>16_	0.031	0.022	0.027	0.000	0.007–0.071	0.016–0.043
F_ROH>32_	0.006	0.012	0.000	0.000	0.000–0.030	0.000–0.013

**Table 3:** Pearson correlation coefficients for all pairs of inbreeding coefficients and proportion of US Brown Swiss genes (BS).

	F_a_Bal_	Ahc	F_a_Kal_	F_New_	F_IS_	F_ROH_	F_ROH>4_	F_ROH>8_	F_ROH>16_	F_ROH>32_	BS
F_PED_	0.498	0.490	0.832	0.964	0.571	0.572	0.567	0.546	0.490	0.383	0.153 ***
F_a_Bal_		0.998	0.803	0.308	0.467	0.395	0.377	0.285	0.166	0.084	0.111 ***
Ahc			0.801	0.297	0.464	0.393	0.376	0.284	0.164	0.085	0.099 ***
F_a_Kal_				0.672	0.559	0.535	0.525	0.466	0.378	0.265	0.150 ***
F_New_					0.516	0.533	0.531	0.530	0.492	0.396	0.143 ***
F_IS_						0.925	0.926	0.851	0.684	0.469	0.070 ***
F_ROH_							0.993	0.936	0.770	0.519	0.070 ***
F_ROH>4_								0.945	0.780	0.526	0.064 **
F_ROH>8_									0.838	0.565	0.049 *
F_ROH>16_										0.681	0.006 ^ns^
F_ROH>32_											−0.027 ^ns^

All Pearson correlation coefficients between inbreeding coefficients were significant with *p*-values < 0.0001. For correlation coefficients with BS: ***: *p*-value < 0.001; **: *p*-value < 0.01; *: *p*-value < 0.05; ns: not significant.

**Table 4:** Least squares means (LSM) and their corresponding standard errors (SE) of average number of ROH, average ROH length and combined for US Brown Swiss classes.

ROH Parameters	BS <60%		BS 60–69%		BS 70–79%		BS 80–89%		BS 90–99%	
	(n = 70)		(n = 228)		(n = 407)		(n = 967)		(n = 636)	
Average number of ROH	33.800	±0.863 ^a^	36.439	±0.478 ^bc^	35.074	±0.358 ^a^	36.070	±0.232 ^c^	37.280	±0.286 ^d^
Average ROH length (Mb)	8.13	±0.14 ^ab^	8.30	±0.08 ^ab^	8.36	±0.06 ^ab^	8.39	±0.04 ^a^	8.24	±0.05 ^b^
Combined ROH length (Mb)	278.86	±9.363 ^a^	304.040	±5.188 ^bc^	294.432	±3.883 ^ab^	303.823	±251.911 ^c^	307.774	±3.106 ^c^

Estimates within rows with different letters are significantly different (*p*-values < 0.05).

**Table 5:** Least squares means (LSM) of F_IS_, F_ROH_, F_ROH>4_, F_ROH>8_, F_ROH>16_, and F_ROH>32_ by US Brown Swiss classes. Corresponding standard errors ranged from 0.001–0.005.

	BS <60%	BS 60–69%	BS 70–79%	BS 80–89%	BS 90–99%
	(n = 70)	(n = 228)	(n = 407)	(n = 967)	(n = 636)
F_IS_	−0.0141 ^a^	0.0003 ^bc^	−0.0041 ^ab^	−0.0019 ^c^	0.0018 ^c^
F_ROH_	0.113 ^a^	0.123 ^bc^	0.119 ^ab^	0.123 ^c^	0.124 ^c^
F_ROH>4_	0.104 ^a^	0.114 ^bc^	0.110 ^ab^	0.114 ^cb^	0.115 ^c^
F_ROH>8_	0.068 ^a^	0.074 ^ab^	0.072 ^ab^	0.075 ^b^	0.075 ^b^
F_ROH>16_	0.028 ^a^	0.032 ^a^	0.031 ^a^	0.033 ^a^	0.031 ^a^
F_ROH>32_	0.006 ^ab^	0.007 ^ab^	0.006 ^ab^	0.007 ^a^	0.005 ^b^

Estimates within rows with different letters (a, b, c) are significantly different (*p*-value < 0.05).

**Table 6:** Least squares means (LSM) and their standard errors (SE) of average number of ROH, average ROH length, F_IS_, F_ROH_, F_ROH>4_, F_ROH>8_, F_ROH>16_, and F_ROH>32_ by survival traits.

	Surv1		Surv3		Surv5		Surv7		Surv9		SE
Homozygosity Item	No	Yes	No	Yes	No	Yes	No	Yes	No	Yes	
	(n = 434)	(n = 1804)	(n = 977)	(n = 791)	(n = 1115)	(n = 329)	(n = 1154)	(n = 273)	(n = 1183)	(n = 243)	
**Average number of ROH**	37.968	34.281 ***	37.427	30.978 ***	37.166	29.032 ***	36.842	29.242 ***	36.595	29.542 ***	0.202–0.454
**Average ROH length (Mb)**	8.413	8.268	8.354	8.221	8.354	8.162 *	8.352	8.141 *	8.360	8.075 ***	0.034–0.075
**F_IS_**	0.011	−0.009 ***	0.008	−0.026 ***	0.006	−0.033 ***	0.004	−0.031 ***	0.002	−0.029 ***	0.001–0.003
**F_ROH_**	0.129	0.115 ***	0.127	0.103 ***	0.126	0.096 ***	0.125	0.097 ***	0.124	0.097 ***	0.001–0.002
**F_ROH>4_**	0.120	0.107 ***	0.117	0.096 ***	0.117	0.090 ***	0.115	0.090 ***	0.115	0.090 ***	0.001–0.002
**F_ROH>8_**	0.079	0.070 ***	0.077	0.062 ***	0.077	0.058 ***	0.076	0.058 ***	0.075	0.058 ***	0.001–0.002
**F_ROH>16_**	0.034	0.029 **	0.033	0.026 ***	0.033	0.024 ***	0.033	0.024 ***	0.032	0.023 ***	0.001–0.002
**F_ROH>32_**	0.007	0.005 *	0.007	0.004 ***	0.007	0.004 ***	0.007	0.003 ***	0.007	0.003 ***	0.000–0.002

Significant differences per homozygosity item and survival trait No and Yes with *p*-values < 0.05: *, with *p*-values < 0.01: **, with *p*-values < 0.001: ***.

**Table 7:** ROH-islands with number of SNPs included, start and end position in bp defined as above the 99th percentile threshold for all animals, by US Brown Swiss classes and by surviving lactation 1 (Surv1), 3 (Surv3), 5 (Surv5), 7 (Surv7), and 9 (Surv9).

Classification	BTA	SNPs	Start	End
All	5	78	74891674	78859007
	6	18	49731100	50316384
	6	281	73932138	91492398
	16	127	21496181	29716390
BS <60%	5	116	74891674	80425933
	6	27	49731100	50746128
	6	239	73932138	90169101
	16	5	25226450	25877452
BS 60–69%	5	112	12308274	24373750
	5	89	74891674	78895966
	6	17	49731100	50291712
	6	222	77603159	90169101
	16	119	21859732	29716390
BS 70–79%	5	117	74162338	80235852
	6	17	49731100	50291712
	6	233	76817878	90169101
	16	115	22445908	29760720
BS 80–89%	5	52	74945315	76888810
	6	27	49731100	50746128
	6	299	73932138	91629835
	16	99	21859732	28688202
BS 90–99%	5	103	72264476	76888810
	6	41	49094600	50746128
	6	220	73932138	90169101
	16	166	21138669	30904995
Surv1	5	53	74891674	76888810
	6	18	49731100	50316384
	6	233	73932138	90169101
	16	127	21496181	29716390
Surv3	5	51	74945315	76888810
	6	17	49731100	50291712
	6	115	82855050	90169101
	16	66	24020699	28259305
Surv5	5	47	75174437	76888810
	6	46	85633295	88134986
Surv7	5	47	75174437	76888810
	6	33	86378938	88134986
Surv9	5	49	75086818	76888810
	6	46	85633295	88134986

## Data Availability

Restrictions apply to the availability of these data. Data were obtained from the Landeskuratorium der Erzeugerringe für tierische Veredelung in Bayern e.V. and the Allgäuer Herdebuchgesellschaft (ProRind) and are available from the authors with the permission of the Landeskuratorium der Erzeugerringe für tierische Veredelung in Bayern e.V. and the Allgäuer Herdebuchgesellschaft (ProRind).

## Supplementary Materials

The following supporting information can be downloaded at: https://www.mdpi.com/article/10.3390/genes15081051/s1, Figure S1: Inbreeding coefficients by birth year; Figure S2: F_ROH_ based on different minimum length by survival classes; Table S1: Survey on genome-wide association studies in Brown Swiss cattle; Table S2: Genomic regions for which significant associations were reported for Brown Swiss cattle populations; Table S3: Studies analyzing ROH islands or selection signatures in Brown Swiss breeds; Table S4: ROH islands, selection signatures and identified candidate genes in US Brown Swiss and Brown cattle breeds with breed proportions of US Brown Swiss; Table S5: Localizations of ROH islands and selection signatures, and identified candidate genes in Original Brown; Table S6: Phenotypic means, standard deviations (SD), and 75% confidence intervals (75% CI) of herd life (HL), length of productive life (LPL), number of calvings (NC), lifetime milk yield (LMY), lifetime fat yield (LFY), lifetime protein yield (LPY), effective lifetime milk yield (EffLMY), effective lifetime fat yield (EffLFY), effective lifetime protein yield (EffLPY), survival to 2nd (Surv1), 4th (Surv3), 6th (Surv5), 8th (Surv7), and 10th (Surv9) lactation, culling rate due to claw and leg disorders (Cul_CL_), infertility (Cul_INF_), and udder diseases (Cul_UD_), proportion of US Brown Swiss and inbreeding coefficients in German Brown cows from all herds (no of cows = 1,032,901) and the 258 herds randomly selected (no of cows = 71,633) by the Allgäuer Herdebuchgesellschaft; Table S7: Inbreeding coefficient F_ROH_ based on different settings for ROH definitions; Table S8: Distribution of SNPs by bovine chromosomes (BTA); Table S9: Parameters for the detection of ROH islands used in previous studies on dairy cattle; Table S10: Means, their standard deviations (SD), standard errors (SE) and 95% confidence limits (CL-95%) of the average number of ROH (N-ROH), average ROH length (Av-ROH), F_IS_, F_ROH_, F_ROH>4_, F_ROH>8_, F_ROH>16_, F_ROH>32_ by US Brown Swiss classes (BS); Table S11: Bootstrap statistics from 10,000 bootstrap samples for the differences of number of ROH (N-ROH), average ROH length (Av-ROH), F_IS_, F_ROH_, F_ROH>4_, F_ROH>8_, F_ROH>16_, and F_ROH>32_ between each two US Brown Swiss classes (BS) with their standard errors (SE), bias and the 95% bootstrap bias-corrected confidence limits (CL-95%); Table S12: Least squares means (LSM) and their corresponding standard errors (SE) of genomic inbreeding coefficients (F_ROH_, F_ROH>4_, F_ROH>8_, F_ROH>16_, and F_ROH>32_) by survival traits and US Brown Swiss classes; Table S13: Least squares means (LSM) and their standard errors (SE) of the average number of ROH, average ROH length, FIS, FROH, FROH>4, FROH>8, FROH>16, and FROH>32 when cows were grouped according to the number of completed lactations (Lact1, Lact2, Lact4, Lact6–Lact8, Lact9–Lact12, and Lact13–Lact17); Table S14a: Consensus ROH shared by at least 30% of the animals; Table S14b: Consensus ROH shared by at least 40% of the animals; Table S14c: Consensus ROH shared by at least 50% of the animals; Table S15: ROH islands with the number of included SNPs (SNPs), start and end position in bp and number of included genes defined as the 99th percentile identified in German Brown; Table S16a: ROH islands with the number of included SNPs (SNPs), start and end position in bp defined as the 95th percentile in German Brown; Table S16b: ROH islands with the number of included SNPs (SNPs), start and end position in bp defined as the 95th percentile by US Brown Swiss classes in German Brown; Table S16c: Comparison of the distribution of the ROH islands of the 95th percentile between US Brown Swiss classes in German Brown; Table S16d: ROH islands on BTA 2, 22 and 28 with the number of included SNPs (SNPs), start and end position in bp defined as the 95th percentile for animals within US Brown Swiss class BS<60%; Table S17a: ROH islands with the number of included SNPs (SNPs), start and end position in bp defined as the 95th percentile by cows surviving the first (Surv1), third (Surv3), fifth (Surv5), seventh (Surv7) and ninth lactation (Surv9); Table S17b: ROH islands on BTA 7 with the number of included SNPs (SNPs), start and end position in bp, number of annotated genes and corresponding gene IDs, defined as the 95th percentile for animals surviving the ninth lactation (Surv9); Table S18: ROH-islands with number of SNPs included, start and end position in bp defined as above the 99th percentile threshold for all animals when cows were grouped according by completed lactation number 2 (Lact2), 4 (Lact4), 6–8 (Lact6–Lact8), 9–12 (Lact9–Lact12) and 13–17 (Lact13–Lact17); Table S19: Panther gene enrichment analysis of ROH islands. Table S20: Quantitative trait loci, which are located within the ROH islands of the 95th percentile threshold and private for specific US Brown Swiss classes. QTL were retrieved from the Animal QTLdb, release 53, a public database developed by a team at the Iowa State University [49].
