# Supplementary material for: Analyzing Runs of Homozygosity Reveals Patterns of Selection in German Brown Cattle
Source: Genes (Basel). 2024 Aug 9;15(8):1051. doi: 10.3390/genes15081051 (PMC11354284; doi:10.3390/genes15081051)
Supplement: Supplementary file 1 [file genes-15-01051-s001.zip › Supplementary Table S1.docx]

**Table S1.** Survey on genome-wide association studies in Brown Swiss cattle.

| Reference | Country | Number of animals | Sex | SNP CHIP | Imputed | Number of SNPs | Method | Phenotypes | Traits |
| --- | --- | --- | --- | --- | --- | --- | --- | --- | --- |
| Guo et al. (2012) [24] | International | 2061 – 5043 (dependend on trait) | Bulls | Illumina Bovine SNP50K Beadchip | no | 44,826 (before QC) | GWAS | DEBV | MY, FY, PY, CRC, ANG, BDE, STA, SCS, MSP |
| Fang and Pausch (2019) [23] | AUT, GER, CH | 4578 | Bulls | Illumina HD Bovine SNP chip (870), Bovine SNP50K v1 or v2 (3708) | yes | 598,016 | GWAS (single and multi-trait) | EBV | MP, BSZ, LC, MGM, FERT, CALV, GQ, CQ |
| Frischknecht et al. (2017) [22] | CH | 1136-4975 (exclusion of animals with OB-prop >0.3) | Bulls | 50 k SNP Chip | yes (2 step) | 9,748,130–9,999,287 sequence variants | GWAS | DEBV | Fertility and calving traits |
| Pausch et al. (2017) [53] |  | 1646 (BV), 6778 (FV), 8805 (HOL) | Bulls | Illumina BovineSNP50K v1 + v2, Illumina Bovine HD | yes | 18,063,587 sequence variants | GWAS, meta-analysis | EBV | FP, PP |
| Dreher et al. (2019) [26] | GER | 1871 | Calves | Illumina BovineSNP50  v2 BeadChip (3072), Illumina Bovine-  HD BeadChip (192) | yes | 525,949 | GWAS |  | Perinatal suckling reflex |
|  |  |  |  |  |  |  |  |  |  |
| Flury et al. (2014) [21] | CH | 1637 | Bulls | Illumina BovineHD  Beadchip, Illumina BovineSNP50k Beadchip | Yes | 624,704 | GWAS | DEBV | Udder traits |
| Häfliger et al. (2021) [25] | CH | 10,085 OB  48,807 BS |  | Different BeadChips | Yes | 114,890 | GWAS, Haplotype analysis | DEBV | Fertility, Birth, growth related traits |
| Widmer et al. (2022) [27] | CH | 3500 OB  7800 BS | Animals |  | Yes | ~600,000 | GWAS, Haplotype analysis | DEBV | Multiple birth |

AUT: Austria, GER: Germany, CH: Switzerland, IT: Italy, MY: millk yield, FY: fat yield, PY: Protein yield, CRC: recycle after calving, ANG: angularity, BDE: body depth, STA: stature, SCS: milk somatic cell, MSP: milking speed, MP: milk production, BS: body size, LC: leg confirmation MGM: mammary gland morphology, FERT: fertility, CALV: calving, GQ: growth, CQ: carcass quality, BS: Brown Swiss, FV: Fleckvieh, HOL: Holstein.
