# Supplementary material for: Analyzing Runs of Homozygosity Reveals Patterns of Selection in German Brown Cattle
Source: Genes (Basel). 2024 Aug 9;15(8):1051. doi: 10.3390/genes15081051 (PMC11354284; doi:10.3390/genes15081051)
Supplement: Supplementary file 1 [file genes-15-01051-s001.zip › Supplementary Table S10_S11.docx]

**Table S10.** Means, their standard deviations (SD), standard errors (SE) and 95% confidence limits (CL-95%) of the average number of ROH (N-ROH), average ROH length (Av-ROH), F_IS_, F_ROH_, F_ROH>4_, F_ROH>8_, F_ROH>16_, F_ROH>32_ by US Brown Swiss classes (BS).

| **Item** | **N-ROH** | **Av-ROH** | **F_IS_** | **F_ROH_** | **F_ROH>4_** | **F_ROH>8_** | **F_ROH>16_** | **F_ROH>32_** |
| --- | --- | --- | --- | --- | --- | --- | --- | --- |
| **BS < 60% (n=70)** |  |  |  |  |  |  |  |  |
| **Mean** | 33.8000 | 8134.6 | -0.01410 | 0.11254 | 0.10401 | 0.06709 | 0.02824 | 0.00577 |
| **SD** | 8.9775 | 1341.2 | 0.05079 | 0.03911 | 0.03778 | 0.03305 | 0.02699 | 0.01266 |
| **SE** | 1.0730 | 160.3 | 0.00607 | 0.00467 | 0.004516 | 0.00395 | 0.00323 | 0.00151 |
| **CL-95%** | 31.6594 35.9406 | 7814.8 8454.4 | -0.02620 -0.00199 | 0.1032 0.1219 | 0.0950 0.1130 | 0.0592 0.0750 | 0.0218 0.0347 | 0.00275 0.00879 |
| **BS 60-69% (n=228)** |  |  |  |  |  |  |  |  |
| **Mean** | 36.4385 | 8303.7 | 0.000298 | 0.12271 | 0.11384 | 0.07400 | 0.031855 | 0.00702 |
| **SD** | 7.3503 | 1214.9 | 0.041816 | 0.03276 | 0.03249 | 0.02874 | 0.02212 | 0.01228 |
| **SE** | 0.4868 | 80.46 | 0.00277 | 0.00217 | 0.00215 | 0.00190 | 0.00146 | 0.00081 |
| **CL-95%** | 35.4794 37.3978 | 8145.1 8462.2 | -0.00516 0.00575 | 0.1184 0.1270 | 0.1096 0.1181 | 0.0703 0.0778 | 0.0290 0.0347 | 0.00542 0.00863 |
| **BS 70-79% (n=407)** |  |  |  |  |  |  |  |  |
| **Mean** | 35.0737 | 8363.1 | -0.00406 | 0.11883 | 0.11015 | 0.07201 | 0.03068 | 0.00596 |
| **SD** | 7.8517 | 1145.65 | 0.04374 | 0.03325 | 0.03267 | 0.02830 | 0.02121 | 0.01169 |
| **SE** | 0.3892 | 56.79 | 0.00217 | 0.00165 | 0.00162 | 0.00140 | 0.00105 | 0.00058 |
| **CL-95%** | 34.3086 35.8388 | 8251.5 8474.8 | -0.00832 0.000207 | 0.1156 0.1221 | 0.1070 0.1133 | 0.0692 0.0748 | 0.0218 0.0347 | 0.00275 0.00879 |
| **BS 80-89% (n=967)** |  |  |  |  |  |  |  |  |
| **Mean** | 36.0703 | 8388.7 | 0.00185 | 0.12262 | 0.11371 | 0.07504 | 0.03229 | 0.00668 |
| **SD** | 7.1404 | 1205.18 | 0.04150 | 0.03202 | 0.03155 | 0.02868 | 0.02287 | 0.01267 |
| **SE** | 0.2296 | 38.76 | 0.00133 | 0.00103 | 0.00102 | 0.00092 | 0.00074 | 0.00041 |
| **CL-95%** | 31.6594 35.9406 | 7814.8 8454.4 | -0.0262 -0.00199 | 0.1032 0.1219 | 0.0950 0.1130 | 0.0592 0.0750 | 0.0218 0.0347 | 0.00275 0.00879 |
| **BS 90-99% (n=636)** |  |  |  |  |  |  |  |  |
| **Mean** | 37.2799 | 8236.7 | 0.00175 | 0.12421 | 0.11483 | 0.07471 | 0.03073 | 0.00531 |
| **SD** | 6.6297 | 1042.2 | 0.03664 | 0.02844 | 0.02815 | 0.02547 | 0.01973 | 0.01033 |
| **SE** | 0.2629 | 41.36 | 0.00145 | 0.00113 | 0.00112 | 0.00101 | 0.00078 | 0.00041 |
| **CL-95%** | 31.6594 35.9406 | 7814.8 8454.4 | -0.0262 -0.00199 | 0.1032 0.1219 | 0.0950 0.1130 | 0.0592 0.0750 | 0.0218 0.0347 | 0.00275 0.00879 |

**Table S11.** Bootstrap statistics from 10,000 bootstrap samples for the differences of number of ROH (N-ROH), average ROH length (Av-ROH), F_IS_, F_ROH_, F_ROH>4_, F_ROH>8_, F_ROH>16_, F_ROH>32_ between each two US Brown Swiss classes (BS) with their standard errors (SE), bias and the 95% bootstrap bias-corrected confidence limits (CL-95%).

|  | **BS <60%** | **BS 60-69%** | **BS 70-79%** | **BS 80-89%** |
| --- | --- | --- | --- | --- |
|  | **SE/Bias/CL-95%** | **SE/Bias/CL-95%** | **SE/Bias/CL-95%** | **SE/Bias/CL-95%** |
| **N-ROH** |  |  |  |  |
| **BS 60-69%** | 1.1675 0.00142  -4.9896 -0.3848 |  |  |  |
| **BS 70-79%** | 1.1317 0.00494  -3.6091 0.8461 | 0.6258 0.00602  0.1418 2.5908 |  |  |
| **BS 80-89%** | 1.1019 -0.00418  -4.4954 -0.1667 | 0.5342 -0.00216 -0.6627 1.4362 | 0.4499 0.00758 -1.8782 -0.1301 |  |
| **BS 90-99%** | 1.1006 -0.0106  -5.6981 -1.3922 | 0.5482 -0.00166 -1.9115 0.2567 | 0.4692 -0.00135 -3.1228 -1.2770 | 0.3511 -0.00407 -1.8872 -0.4973 |
| **Av-ROH** |  |  |  |  |
| **BS 60-69%** | 179.3 -0.1562  -511.3 - 194.9 |  |  |  |
| **BS 70-79%** | 168.4 -0.2559 -539.9 119.8 | 97.6674 0.1684 -247.1 134.2 |  |  |
| **BS 80-89%** | 165.1 -0.8346 -559.1 98.2741 | 88.5681 0.3970 -255.5 94.6049 | 69.0402 0.3394 -158.7 111.7 |  |
| **BS 90-99%** | 163.3 1.6893 -407.6 237.3 | 90.1241 -0.2729 -107.8 246.6 | 69.4138 -0.6870 -5.2171 264.7 | 56.5327 -0.0380 42.5001 262.8 |
| **F_IS_** |  |  |  |  |
| **BS 60-69%** | 0.00656 -0.00004 -0.0276 -0.00158 |  |  |  |
| **BS 70-79%** | 0.00644 -0.00003 -0.0224 0.00260 | 0.00351 -0.00002 -0.00249 0.0112 |  |  |
| **BS 80-89%** | 0.00619 0.000102 -0.0281 -0.00395 | 0.00306 -0.00006 -0.00758 0.00449 | 0.00252 0.000017 -0.0109 -0.00091 |  |
| **BS 90-99%** | 0.00624 0.000022 -0.0282 -0.00364 | 0.00313 -0.00005 -0.00758 0.00463 | 0.00264 0.000022 -0.0110 -0.00067 | 0.00195 -3.61E-6 -0.00376 0.00387 |
| **F_ROH_** |  |  |  |  |
| **BS 60-69%** | 0.00518 -0.00011 -0.0201 0.000169 |  |  |  |
| **BS 70-79%** | 0.00489 -0.00009 -0.0155 0.00358 | 0.00272 -6.66E-6 -0.00147 0.00933 |  |  |
| **BS 80-89%** | 0.00474 0.000032 -0.0195 -0.00107 | 0.00238 9.885E-7 -0.00475 0.00470 | 0.00195 0.000017 -0.00753 0.000135 |  |
| **BS 90-99%** | 0.00482 0.000026 -0.0209 -0.00190 | 0.00246 0.000067 -0.00638 0.00328 | 0.00200 -0.00002 -0.00928 -0.00142 | 0.00153 0.000018 -0.00462 0.00136 |
| **F_ROH>4_** |  |  |  |  |
| **BS 60-69%** | 0.00496 -0.00009 -0.0192 0.000156 |  |  |  |
| **BS 70-79%** | 0.00472 -0.00009 -0.0153 0.00344 | 0.00269 0.000016 -0.00145 0.00909 |  |  |
| **BS 80-89%** | 0.00463 -0.00001 -0.0185 -0.00016 | 0.00238 5.507E-6 -0.00453 0.00481 | 0.00191 -0.00002 -0.00727 0.000217 |  |
| **BS 90-99%** | 0.00461 -0.00002 -0.0195 -0.00150 | 0.00242 -0.00002 -0.00556 0.00387 | 0.00194 -1.17E-6 -0.00842 -0.00084 | 0.00151 0.000013 -0.00415 0.00184 |
| **F_ROH>8_** |  |  |  |  |
| **BS 60-69%** | 0.00439 0.000045 -0.0152 0.00217 |  |  |  |
| **BS 70-79%** | 0.00414 0.000031 -0.0125 0.00366 | 0.00235 1.584E-7 -0.00259 0.00671 |  |  |
| **BS 80-89%** | 0.00327 -0.00004 -0.00958 0.00350 | 0.00213 -0.00002 -0.00507 0.00316 | 0.00169 0.000013 -0.00634 0.000199 |  |
| **BS 90-99%** | 0.00407 -0.00004 -0.0151 0.000960 | 0.00216 0.000067 -0.00493 0.00347 | 0.00174 -0.00002 -0.00603 0.000743 | 0.00137 0.000013 -0.00241 0.00293 |
| **F_ROH>16_** |  |  |  |  |
| **BS 60-69%** | 0.00355 8.893E-6 -0.00981 0.00422 |  |  |  |
| **BS 70-79%** | 0.00333 -6.11E-6 -0.00822 0.00497 | 0.00178 -0.00005 -0.00221 0.00476 |  |  |
| **BS 80-89%** | 0.00406 0.000010 -0.0154 0.000618 | 0.00163 -6.9E-6 -0.00350 0.00296 | 0.00129 0.000015 -0.00415 0.000897 |  |
| **BS 90-99%** | 0.00331 0.000046 -0.00803 0.00513 | 0.00167 0.000019 -0.00208 0.00449 | 0.00129 0.000016 -0.00261 0.00248 | 0.00108 0.000013 -0.00056 0.00367 |
| **F_ROH>32_** |  |  |  |  |
| **BS 60-69%** | 0.00171 -0.00003 -0.00429 0.00251 |  |  |  |
| **BS 70-79%** | 0.00163 -2.14E-6 -0.00301 0.00342 | 0.000999 -0.00001 -0.00082 0.00309 |  |  |
| **BS 80-89%** | 0.00155 -7.2E-6 -0.00363 0.00260 | 0.000915 6.496E-6 -0.00137 0.00222 | 0.000710 0.000011 -0.00211 0.000665 |  |
| **BS 90-99%** | 0.00157 0.000015 -0.00222 0.00403 | 0.000903 -1.35E-6 0.000045 0.00358 | 0.000713 9.268E-6 -0.00073 0.00206 | 0.000577 -6.41E-6 0.000228 0.00249 |
