# Supplementary material for: Analyzing Runs of Homozygosity Reveals Patterns of Selection in German Brown Cattle
Source: Genes (Basel). 2024 Aug 9;15(8):1051. doi: 10.3390/genes15081051 (PMC11354284; doi:10.3390/genes15081051)
Supplement: Supplementary file 1 [file genes-15-01051-s001.zip › Supplementary Table S13.docx]

**Table S13.** Least squares means (LSM) and their standard errors (SE) of the average number of ROH, average ROH length, FIS, FROH, FROH>4, FROH>8, FROH>16, FROH>32 when cows were grouped according to the number of completed lactations (Lact1, Lact2, Lact4, Lact6-Lact8, Lact9-Lact12, Lact13-Lact17).

| **Item** | **Lact1** | **Lact2** | **Lact4** | **Lact6-Lact8** | **Lact9-Lact12** | **Lact13-Lact17** | **SE** |
| --- | --- | --- | --- | --- | --- | --- | --- |
| **n** | 434 | 494 | 331 | 64 | 196 | 69 |  |
| **Average number of ROH** | 37.975 | 38.083 | 36.921 | 29.609 | 29.327 | 28.565 | 0.290-0.826 |
| **Average ROH length (Mb)** | 8417 | 8336 | 8325 | 8451 | 8221 | 7854 | 51-145 |
| **F_IS_** | 0.0115 | 0.0108 | 0.0017 | -0.0427 | -0.0298 | -0.0327 | 0.002-0.005 |
| **F_ROH_** | 0.1294 | 0.1286 | 0.1241 | 0.1016 | 0.0983 | 0.0917 | 0.001-0.005 |
| **F_ROH>4_** | 0.1199 | 0.1193 | 0.1147 | 0.0940 | 0.0912 | 0.0844 | 0.001-0.004 |
| **F_ROH>8_** | 0.0793 | 0.0783 | 0.0747 | 0.0624 | 0.0592 | 0.0536 | 0.001-0.003 |
| **F_ROH>16_** | 0.0343 | 0.0326 | 0.0319 | 0.0287 | 0.0248 | 0.0196 | 0.001-0.003 |
| **F_ROH>32_** | 0.0073 | 0.0064 | 0.0058 | 0.0059 | 0.0040 | 0.0018 | 0.001 |
