# Supplementary material for: Analyzing Runs of Homozygosity Reveals Patterns of Selection in German Brown Cattle
Source: Genes (Basel). 2024 Aug 9;15(8):1051. doi: 10.3390/genes15081051 (PMC11354284; doi:10.3390/genes15081051)
Supplement: Supplementary file 1 [file genes-15-01051-s001.zip › Supplementary Table S15_all99genes.docx]

**Table S15.** ROH islands with the number of included SNPs (SNPs), start and end position in bp and number of included genes defined as the 99th percentile identified in German Brown.

| **BTA** | **SNPs** | **Start** | **End** | **No of**  **genes** | **Gene ID** |
| --- | --- | --- | --- | --- | --- |
| 5 | 78 | 74891674 | 78859007 | 46 | *TXN2, FOXRED2, EIF3D, CACNG2, IFT27, PVALB, NCF4, CSF2RB, TEX33, TST, MPST, KCTD17, TMPRSS6, IL2RB, CIQTNF6, SSTR3, RAC2, bta-mir-1835, CYTH4, ELFN2, MFNG, CARD10, U6, U6, USP18, ALG10, SYT10, PKP2, YARS2, DNM1L, FGD4, U6, BICD1, RESF1, AMN1, ETFBKMT, U6, DENND5B, SINHCAF, CAPRIN2, IPO8* |
| 6 | 18 | 49731100 | 50316384 | 1 | *PCDH7* |
| 6 | 281 | 73932138 | 91492398 | 123 | *Y RNA, ADGRL3, 5S rRNA, TECRL, EPHA5, CENPC, STAP1, UBA6, GNRHR, TMPRSS11D, TMPRSS11A, TMPRSS11F, TMPRSS11BNL, NAP1L1, TMPRSS11E, 5S rRNA, U6, YTHDC1, UGT2B10, 5S rRNA, MGC152010, UGT2A1, SULT1B1, SULT1E1, CSN1S1, CSN2, HSTN, CSN1S2, ODAM, CSN3, CABS1, AMTN, AMBN, ENAM, JCHAIN, UTP3, RUFY3, GRSF1, MOB1B, DCK, SLC4A4, GC, NPFFR2, ADAMTS3, SNORD42, COX18, ANKRD17, ALB, AFP, AFM, RASSF, 7SK, CXCL8, CXCL5, CXCL2, CXCL3, GRO1, MTHFD2L, EPGN, EREG, AREG, BTC, PARM1, RCHY1, THAP6, ODAPH, CDKL2, G3BP2, USO1, U1, PPEF2, NAAA, SDAD1, CXCL9, ART3, CXCL10, CXCL11, NUP54, SCARB2, STBD1, CCDC158, U6, SHROOM3* |
| 16 | 127 | 21496181 | 29716390 | 67 | *RRP15, TGFB2, U6, LYPLAL1, SLC30A10, EPRS1, BPNT1, IARS2, bta-mir-215, bta-mir-194-1, RAB3GAP2, SNORA36B, bta-mir-664b, MARK1, C16H1orf115, MTARC2, MTARC1, HLX, DUSP10, U8, HHIPL2, TAF1A, MIA3, AIDA, BROX, FAM177B, DISP1, TLR5, SUSD4, CCDC185, CAPN8, CAPN2, TP53BP2, FBXO28, DEGS1, NVL, CNIH4, WDR26, CNIH3, DNAH14, LBR, ENAH, SRP9, EPHX1, TMEM63A, LEFTY2, PYCR2, SDE2, H3-3A, ACBD3, MIXL1, LIN9, PARP1, STUM, ITPKB* |
