# Supplementary material for: Analyzing Runs of Homozygosity Reveals Patterns of Selection in German Brown Cattle
Source: Genes (Basel). 2024 Aug 9;15(8):1051. doi: 10.3390/genes15081051 (PMC11354284; doi:10.3390/genes15081051)
Supplement: Supplementary file 1 [file genes-15-01051-s001.zip › Supplementary Table S16d.docx]

**Table S16d.** ROH islands on BTA 2, 22, and 28 with the number of included SNPs (SNPs), start and end position in bp defined as the 9*5*th percentile for animals within US Brown Swiss class BS<60%.

| BTA | SNPs | Start | End | No of Genes | Gene ID |
| --- | --- | --- | --- | --- | --- |
| 2 | 115 | 76255186 | 81737952 | 10 | ***CNTNAP5****,* ***GYPC****, GLS,* ***STAT1****, STAT4, MYO1B, NABP1, CAVIN2, TMEFF2, U6* |
| 22 | 310 | 12008710 | 31409453 | 124 | *ZNF660, ZNF197, ZNF35, ZNF502, KIAA1143, KIF15, TATDN2, IRAK2, VHL, BRK1, FANCD2OS, FANCD2, EMC3, , PRRT3, CRELD1, IL17RC, IL17RE, JAGN1, CIDEC, RPUSD3, TTLL3, ARPC4, TADA3, CAMK1, OGG1, BRPF1, CPNE9, MTMR14, , U6, LHFPL4, SETD5, THUMPD3, SRGAP3, RAD18, OXTR, CAV3, SSUH2, , LMCD1, GRM7, U6, U6, 5S_rRNA, EDEM1, ARL8B, BHLHE40****, ITPR1****, , bta-mir-2285am, U6, SUMF1, , SETMAR, , LRRN1, , CRBN, TRNT1, IL5RA, CNTN4, CNTN6, U6, CHL1, , U6,* ***CNTN3****, PDZRN3, PPP4R2, GXYLT2, SHQ1, , RYBP, , PROK2, GPR27, EIF4E3, , bta-mir-1284, , FOXP1, 5S_rRNA, U6, MDFIC2* |
| 28 | 139 | 15972452 | 23434162 | 27 | *U6, CDK1,* ***RHOBTB1****, U6, TMEM26, CABCOCO1,* ***ARID5B****, RTKN2, ZNF365, ADO, EGR2, NRBF2, JMJD1C, MIR1296, REEP3, 7SK, U6,* ***CTNNA3****, LRRTM3* |
