# Supplementary material for: Analyzing Runs of Homozygosity Reveals Patterns of Selection in German Brown Cattle
Source: Genes (Basel). 2024 Aug 9;15(8):1051. doi: 10.3390/genes15081051 (PMC11354284; doi:10.3390/genes15081051)
Supplement: Supplementary file 1 [file genes-15-01051-s001.zip › Supplementary Table S17_Surv.docx]

**Table S17a**. ROH islands with the number of included SNPs (SNPs), start and end position in bp defined as the 95th percentile for animals surviving the first (Surv1), third (Surv3), fifth (Surv5), seventh (Surv7), and ninth lactation (Surv9).

|  | **BTA** | **SNPs** | **Start** | **End** |
| --- | --- | --- | --- | --- |
| **Surv1** | | | | |
|  | 4 | 97 | 5212181 | 10319594 |
|  | 5 | 263 | 10365435 | 27670995 |
|  | 5 | 142 | 32428588 | 40618209 |
|  | 5 | 353 | 70651696 | 90614584 |
|  | 5 | 37 | 94576821 | 96995660 |
|  | 6 | 5 | 16407876 | 16681533 |
|  | 6 | 6 | 28366514 | 29062262 |
|  | 6 | 119 | 36441375 | 40928447 |
|  | 6 | 257 | 43579172 | 56589533 |
|  | 6 | 87 | 58077967 | 62594145 |
|  | 6 | 8 | 70246689 | 70531796 |
|  | 6 | 426 | 72945112 | 97072470 |
|  | 11 | 16 | 31369325 | 32068438 |
|  | 11 | 100 | 64307937 | 69532252 |
|  | 11 | 17 | 88557682 | 91627358 |
|  | 12 | 36 | 62971636 | 67565619 |
|  | 12 | 116 | 78500125 | 85195005 |
|  | 16 | 216 | 19974892 | 31798443 |
|  | 19 | 153 | 45297607 | 52552004 |
|  | 20 | 79 | 21658212 | 26352669 |
| **Surv3** | | | | |
|  | 4 | 53 | 7372107 | 10319594 |
|  | 4 | 1 | 15667206 | 15667206 |
|  | 5 | 40 | 11670209 | 14727426 |
|  | 5 | 106 | 20051652 | 27324084 |
|  | 5 | 103 | 32428588 | 39468760 |
|  | 5 | 281 | 70651696 | 85407207 |
|  | 5 | 34 | 95028489 | 96995660 |
|  | 6 | 134 | 35594486 | 40928447 |
|  | 6 | 252 | 43579172 | 56589533 |
|  | 6 | 82 | 58077967 | 62338657 |
|  | 6 | 8 | 70246689 | 70531796 |
|  | 6 | 416 | 73770751 | 97039335 |
|  | 11 | 99 | 64352461 | 69532252 |
|  | 11 | 124 | 88557682 | 97758258 |
|  | 12 | 6 | 62971636 | 63111014 |
|  | 12 | 91 | 79751803 | 84624747 |
|  | 16 | 205 | 20297366 | 31704359 |
|  | 19 | 128 | 45297607 | 51347919 |
|  | 20 | 52 | 22523592 | 25422359 |
| **Surv5** | | | | |
|  | 5 | 281 | 70651696 | 85407207 |
|  | 6 | 105 | 36125455 | 40130949 |
|  | 6 | 112 | 46751512 | 51441525 |
|  | 6 | 31 | 59716766 | 61390096 |
|  | 6 | 398 | 73770751 | 96178236 |
|  | 11 | 94 | 64576376 | 69532252 |
|  | 11 | 148 | 88853103 | 97758258 |
|  | 12 | 20 | 62971636 | 63738585 |
|  | 16 | 180 | 21138669 | 31587509 |
|  | 19 | 75 | 48149532 | 51347919 |
| **Surv7** | | | | |
|  | 5 | 281 | 70651696 | 85407207 |
|  | 6 | 106 | 36441375 | 40808612 |
|  | 6 | 112 | 46751512 | 51441525 |
|  | 6 | 41 | 59716766 | 61390096 |
|  | 6 | 371 | 73770751 | 95406549 |
|  | 11 | 94 | 64576376 | 69532252 |
|  | 11 | 148 | 88853103 | 97758258 |
|  | 11 | 4 | 99831957 | 99932332 |
|  | 12 | 6 | 62971636 | 63111014 |
|  | 16 | 184 | 20331005 | 31704359 |
|  | 19 | 75 | 48149532 | 51347919 |
|  | 20 | 30 | 22651571 | 25160596 |
| **Surv9** | | | | |
|  | 5 | 281 | 70651696 | 85407207 |
|  | 6 | 115 | 36125455 | 40808612 |
|  | 6 | 127 | 45935611 | 51441525 |
|  | 6 | 41 | 59716766 | 61390096 |
|  | 6 | 367 | 73932138 | 95345674 |
|  | 7 | 35 | 42804161 | 45102787 |
|  | 11 | 94 | 64576376 | 69532252 |
|  | 11 | 173 | 88853103 | 99932332 |
|  | 12 | 14 | 62971636 | 63357328 |
|  | 16 | 193 | 20297366 | 31704359 |
|  | 19 | 75 | 48149532 | 51347919 |
|  | 20 | 4 | 24862047 | 25160596 |

**Colour legend: private for Surv1, private for Surv9, common for Surv1 and Surv3.**

**Table S17b.** ROH islands on BTA 7 with the number of included SNPs (SNPs), start and end position in bp, number of annotated genes and corresponding gene IDs, defined as the 9*5*th percentile for animals surviving lactation 9 (Surv9).

| **SNPs** | **Start** | **End** | **No of genes** | **Gene ID** |
| --- | --- | --- | --- | --- |
| 35 | 42804161 | 45102787 | 86 | *OR2AV2, OR2AV11, OR2AZ3, OR2AZ3B, OR2AZ1, PLPP2, MIER2, THEG, C2CD4C, SHC2, ODF3L2, , MADCAM1, TPGS1, CDC34, GZMM, BSG, HCN2, POLRMT, FGF22, RNF126, FSTL3, PRSS57, PALM, MISP, PTBP1, PLPPR3, AZU1, U6, PRTN3, ELANE, CFD, MED16, U6, R3HDM4, KISS1R, ARID3A, WDR18, GRIN3B, TMEM259, CNN2, ABCA7, ARHGAP45, POLR2E, GPX4, SBNO2, STK11, CBARP, ATP5F1D, MIDN, CIRBP, FAM174C, EFNA2, PWWP3A, NDUFS7, GAMT, DAZAP1, RPS15, APC2, C7H19orf25, PCSK4, REEP6, , ADAMTSL5, , MEX3D, MBD3, UQCR11, TCF3, ONECUT3, ATP8B3, REXO1, KLF16, ABHD17A, , SCAMP4, CSNK1G2, bta-mir-6120, BTBD2, SOWAHA, SHROOM1, GDF9, UQCRQ, LEAP2, AFF4, U6, ZCCHC10, HSPA4, 5S_rRNA, FSTL4* |
