# Supplementary material for: Analyzing Runs of Homozygosity Reveals Patterns of Selection in German Brown Cattle
Source: Genes (Basel). 2024 Aug 9;15(8):1051. doi: 10.3390/genes15081051 (PMC11354284; doi:10.3390/genes15081051)
Supplement: Supplementary file 1 [file genes-15-01051-s001.zip › Supplementary Table S18.docx]

**Table S18.** ROH-islands with number of SNPs included, start and end position in bp defined as above the 99^th^ percentile threshold for all animals when cows were grouped according by completed lactation number 2 (Lact2), 4 (Lact4), 6-8 (Lact6-Lact8), 9-12 (Lact9-Lact12) and 13-17 (Lact13-Lact17).

| **Classification** | **BTA** | **SNPs** | **Start** | **End** |
| --- | --- | --- | --- | --- |
| Lact2 | 5 | 95 | 73063778 | 78758040 |
|  | 6 | 17 | 49731100 | 50291712 |
|  | 6 | 239 | 73932138 | 90169101 |
|  | 16 | 127 | 21496181 | 29716390 |
| Lact4 | 5 | 55 | 74945315 | 76888810 |
|  | 6 | 57 | 48120650 | 50746128 |
|  | 6 | 264 | 73932138 | 91492398 |
|  | 16 | 128 | 21496181 | 29760720 |
| Lact6-Lact8 | 5 | 127 | 73992231 | 78895966 |
|  | 6 | 39 | 36983415 | 38157637 |
|  | 6 | 299 | 73976015 | 91629835 |
| Lact9-Lact12 | 5 | 47 | 75174437 | 76888810 |
|  | 6 | 46 | 85633295 | 88134986 |
| Lact13-Lact17 | 5 | 52 | 74945315 | 76888810 |
|  | 6 | 39 | 36635943 | 38157637 |
|  | 6 | 13 | 86924174 | 87590471 |
|  | 16 | 25 | 24174300 | 26114830 |
