# Supplementary material for: Analyzing Runs of Homozygosity Reveals Patterns of Selection in German Brown Cattle
Source: Genes (Basel). 2024 Aug 9;15(8):1051. doi: 10.3390/genes15081051 (PMC11354284; doi:10.3390/genes15081051)
Supplement: Supplementary file 1 [file genes-15-01051-s001.zip › Supplementary Table S3.docx]

**Table S3.** Studies analyzing ROH islands or selection signatures in Brown Swiss breeds.

| Reference | Breed | Number of animals | Sex | SNP CHIP | Imputed | Number of SNPs | Method |  |  |
| --- | --- | --- | --- | --- | --- | --- | --- | --- | --- |
| Cesarani et al. (2018) [19] | Italian Brown Swiss | 22 | Animals | Illumina BovineSNP50 | Yes | 43012 | ROH-Island |  |  |
| Lozada-Soto et al. (2022) [52] | US Brown Swiss | 39,040 | Animals | - | - | 62,564 | ROH-Island | 0.995 percentile |  |
| Moscarelli et al. (2021) [18] | Swiss Original Brown, German Brown (Original) | 55 | Animals | Bovine  SNP50K BeadChip |  | 34,735 | ROH-Island | 0.999 percentile |  |
|  | US Brown Swiss, Italian Brown Swiss (modern) | 51 | Animals | Bovine  SNP50K BeadChip |  | 34,735 | ROH-Island | 0.999 percentile |  |
| Signer-Hasler et al. (2017) [29] | US Brown Swiss, Swiss Brown, Swiss Original Brown (and 6 further breeds) | 281, 3386, 167 (+ 5380) | Bulls | Bovine  SNP50K BeadChip | No | 27,612 | Selection signatures | F_ST_ |  |
| Rothammer et al. (2013) [30] | Original Brown, German Brown Swiss (and other breeds) | 48, 568 (+ 1224) | Animals | BovineSNP50 | No | 47,651 | Selection signatures | XP-EHH |  |
| Bhati et al. (2020) [31] | Swiss Original Brown | 49 | Bulls | Illumina  HiSeq 2500 (30) or Illumina HiSeq 4000 (19) |  | 15,722,811 SNPs, 1580878 Indels | Selection signatures | iHS  CLR |  |

iHS: integrated haplotype score, CLR: Composite likelihood ratio, XP-EHH: cross population extended haplotype homozygosity.
