# Supplementary material for: Analyzing Runs of Homozygosity Reveals Patterns of Selection in German Brown Cattle
Source: Genes (Basel). 2024 Aug 9;15(8):1051. doi: 10.3390/genes15081051 (PMC11354284; doi:10.3390/genes15081051)
Supplement: Supplementary file 1 [file genes-15-01051-s001.zip › Supplementary Table S4.docx]

**Table S4.** ROH islands, selection signatures and identified candidate genes in US Brown Swiss and Brown cattle breeds with breed proportions of US Brown Swiss.

| Reference | N |  | Breed | Method | BTA | Position | Assembly | Associated Genes | Effects proposed on |
| --- | --- | --- | --- | --- | --- | --- | --- | --- | --- |
| Signer-Hasler et al. (2017)^1^ [29] |  |  | US-BS | F_ST_ | 5 | 76,432,958 – 78,432,958 |  | SYT | Longevity |
|  |  |  | SBV | F_ST_ | 6 | 32,407,621 – 34,407,621 |  |  |  |
|  |  |  | US-BS, SBV | F_ST_ | 16 | 24,736,714 – 26,736,714 |  | HLX | Feed efficiency |
|  |  |  | US-BS | F_ST_ | 16 | 26,450,435 – 28,450,435 |  | TLR5, CAPN8, CAPN2 | Disease resistance, meat quality |
| Moscarelli et al. (2021) [18] |  |  | US-BS + ItBV | ROH-Island | 5 | 75,086,818 – 78,560,464 | ARS-UCD 1.2 | CACNG2, IFT27, LOC101906363, LOC101906435, PVALB,  LOC107132513**, NCF4**, **CSF2RB**, LOC788541, TEX33, TST, **MPST**,  KCTD17, **TMPRSS6**, IL2RB, LOC510185**, C1QTNF6**, **SSTR3, RAC2**,  MIR1835, CYTH4, LOC107132508, ELFN2, LOC107132514, MFNG,  CARD10, USP18, ALG10, SYT10, LOC107132509, PKP2, YARS2,  DNM1L, LOC101907810, FGD4, BICD1, LOC782092, KIAA1551,  LOC100137780, LOC104972515, AMN1, ETFBKMT, DENND5B | Milk production, |
|  |  |  | US-BS + ItBV | ROH-Island | 5 | 79,334,574 – 80,235,852 |  | TMTC1, LOC518980, ERGIC2 |  |
|  |  |  | US-BS + ItBV | ROH-Island | 6 | 86,399,795 – 87,818,793 |  | LOC112447099, **SLC4A4**, LOC782958, **GC, NPFFR2, ADAMTS3**, TRNACGCA | Mastitis, Longevity |
| Rothammer et al. (2013) [30] |  |  | GBV | XP-EHH | 6 | 84,205,130 -  96,165,559 | UMD 3.1 | BTC, ANKRD17, CSN1S1, CSN2, CSN1S2, CSN3, IL8 | Casein cluster |
|  |  |  | GBV | XP-EHH | 6 | 37,897,068-38,052,662 |  | ABCG2 | Production |
|  |  |  | GBV | XP-EHH | 19 | 47,693,674-51,182,163 |  | GH1 | Production |
| Cesarani et al. (2018) [19] |  |  | ItBV | ROH-island | 1 | 103.5 – 105.5 Mb | UMD 3.1 |  |  |
|  |  |  | ItBV | ROH-island | 6 | 30 – 40 Mb |  | ABCG2, SPP1, LCORL, NCAPG |  |
|  |  |  | ItBV | ROH-island | 6 | 80 – 95 Mb |  | Casein cluster |  |
|  |  |  | ItBV | ROH-island | 20 | 14 – 25 Mb |  | IPO11 |  |
| Lozada-Soto et al. (2022) [52] |  |  | US-BS | ROH-island | 5 | 75,246,880 – 76,724,612 | ARS-UCD 1.2 | ENSBTAG00000007531 ENSBTAG00000009064 ENSBTAG00000030652 ENSBTAG00000030650 ENSBTAG00000030648 ENSBTAG00000030646 ENSBTAG00000032152 ENSBTAG00000016345 ENSBTAG00000008074 ENSBTAG00000008910 ENSBTAG00000011043 ENSBTAG00000014237 ENSBTAG00000007259 ENSBTAG00000015043 ENSBTAG00000015044 ENSBTAG00000016661 ENSBTAG00000030632 ENSBTAG00000000606 |  |
|  |  |  | US-BS | ROH-island | 6 | 85,662,466 – 91,174,173 |  | ENSBTAG00000019849 ENSBTAG00000002928 ENSBTAG00000004793 ENSBTAG00000010346 ENSBTAG00000018531 ENSBTAG00000055134 ENSBTAG00000009310 ENSBTAG00000016795 ENSBTAG00000008577 ENSBTAG00000051236 ENSBTAG00000016290 ENSBTAG00000012397 ENSBTAG00000002348 ENSBTAG00000013718 ENSBTAG00000049290 ENSBTAG00000009070 ENSBTAG00000006507 ENSBTAG00000005394 ENSBTAG00000004912 ENSBTAG00000017121 ENSBTAG00000017131 ENSBTAG00000047833 ENSBTAG00000049436 ENSBTAG00000011935 ENSBTAG00000019716 ENSBTAG00000027534 ENSBTAG00000009812 ENSBTAG00000011961 ENSBTAG00000027513 ENSBTAG00000051891 ENSBTAG00000037778 ENSBTAG00000037558 ENSBTAG00000043960 ENSBTAG00000004052 ENSBTAG00000010273 ENSBTAG00000018134 ENSBTAG00000004237 ENSBTAG00000015919 ENSBTAG00000007189 ENSBTAG00000007190 ENSBTAG00000050403 ENSBTAG00000046788 ENSBTAG00000014038 ENSBTAG00000014041 ENSBTAG00000017028 ENSBTAG00000015449 ENSBTAG00000019478 ENSBTAG00000007639 ENSBTAG00000050852 ENSBTAG00000010954 ENSBTAG00000001725 ENSBTAG00000005603 ENSBTAG00000010955 ENSBTAG00000010956 ENSBTAG00000053885 |  |
|  |  |  | US-BS | ROH-island | 16 | 22,866,782 – 28,123,691 |  | ENSBTAG00000004414 ENSBTAG00000006898 ENSBTAG00000004064 ENSBTAG00000012582 ENSBTAG00000012585 ENSBTAG00000010460 ENSBTAG00000048856 ENSBTAG00000053321 ENSBTAG00000016277 ENSBTAG00000047287 ENSBTAG00000014560 ENSBTAG00000001729 ENSBTAG00000017561 ENSBTAG00000013483 ENSBTAG00000018824 ENSBTAG00000007593 ENSBTAG00000007595 ENSBTAG00000049754 ENSBTAG00000049277 ENSBTAG00000049777 ENSBTAG00000000477 ENSBTAG00000020989 ENSBTAG00000006704 ENSBTAG00000003600 ENSBTAG00000012778 ENSBTAG00000012781 ENSBTAG00000019538 ENSBTAG00000012705 ENSBTAG00000006718 ENSBTAG00000019471 ENSBTAG00000011785 ENSBTAG00000053199 ENSBTAG00000008449 |  |
|  |  |  |  |  |  |  |  |  |  |

^1^di-value >10, ItBV: Italian Brown (Italienisches Braunvieh), GBV: German Brown (Deutsches Braunvieh), SBV: Swiss Brown (Schweizer Braunvieh), US-BS: US Brown Swiss
