# Supplementary material for: Analyzing Runs of Homozygosity Reveals Patterns of Selection in German Brown Cattle
Source: Genes (Basel). 2024 Aug 9;15(8):1051. doi: 10.3390/genes15081051 (PMC11354284; doi:10.3390/genes15081051)
Supplement: Supplementary file 1 [file genes-15-01051-s001.zip › Supplementary Table S5.docx]

**Table S5.** Localisations of ROH islands and selection signatures, and identified candidate genes in Original Brown.

| Reference | N | |  | Method | BTA | | Position | Assembly | | Associated Genes | Trait |
| --- | --- | --- | --- | --- | --- | --- | --- | --- | --- | --- | --- |
| Signer-Hasler et al. (2017)^1^ [29] | 167 | |  | F_ST_ | 11 | 65,512,895 – 67,512,895 | |  | LOC786621,ETAA1,LOC104973406,LOC101904865,C1D,LOC104973407,WDR92,PNO1,  PPP3R1,LOC104973408,CNRIP1,LOC104973409,PLEK,FBXO48,APLF,**PROKR1**,ARHGAP25,  BMP10,LOC509961,LOC104973410,GKN2,GKN1,ANTXR1 | | Fertility |
|  | 167 | |  | F_ST_ | 11 | 67,608,705 – 69,608,705 | |  | GFPT1,NFU1,AAK1,LOC787229,LOC104973411,ANXA4,GMCL1,SNRNP27,MXD1,ASPRV1,  LOC104973412,LOC101905499,**PCBP1**,LOC104973413,C11H2orf42,TIA1,TRNAG-CCC,PCYOX1,SNRPG,**EHD3**,**CAPN14**,LOC104973414,GALNT14,**CAPN13,**LOC101905676,  LCLAT1,LOC104973415,LBH,LOC104973416 | | Fertility, meat quality |
|  |  | |  | F_ST_ | 14 | 23,392,111- 25,392,111 | |  | OPRK1,ATP6V1H,RGS20,TCEA1,LYPLA1,MRPL15,LOC101906669,TRNAG-UCC,TRNAC-GCA,SOX17,TRNAG-CCC,RP1,LOC104974018,XKR4,TRNAT-AGU,TMEM68,**TGS1**,LOC104974019**,LYN**,RPS20,MOS,**PLAG1,CHCHD7**,SDR16C5,  SDR16C6,PENK,LOC101907667 | | Fertility, stature |
| Rothammer et al. (2013) [30] | | 35 |  | XP-EHH | 6 | 37,642,516-38,052,662 | | UMD 3.1 | ABCG2 | |  |
|  | 35 | |  | XP-EHH | 14 | 1-2,228,124 | |  | DGAT1 | |  |
|  | 35 | |  | XP-EHH | 11 | 64,291,486 - 71,493,179 | |  | PROKR1, GFPT1, GMCL1, PCBP1, EHD3 | | Fertility |
| Bhati et al. (2020)^2^ [31] | 49 | |  | CLR | 11 | 66,019,842 -  68,540,358 | | UMD 3.1 | AAK1, NFU1, ANXA4, NFU1 | |  |
|  | 49 | |  | iHS | 11 | 68,446,304 – 69,286,304 | |  | D11H2orf42, PCYOX1, TIA1, CAPN14, GALNT14, CAPN13 | |  |
|  | 49 | |  | CLR | 6 | 38,537,957 -  39,458,373 | |  | DCAF16,NCAPG,FAM184B,LAP3, MED28LCORL,NCAPG | | Pre-and postnatal growth, Calving difficulties, milk production |
| Moscarelli et al. (2021) [18] | | 55 |  | ROH island 0.995th per-centile | 11 | 65,681,864 -  71,263,018 | | ARS-UCD 1.2 | ETAA1, LOC101904865, **C1D, WDR92, PNO1, PPP3R1**, CNRIP1,LOC104973409, **PLEK**, FBXO48, APLF, PROKR1, **ARHGAP25**, BMP10,LOC509961, LOC104973410, GKN2,GKN1,**ANTXR1**,GFPT1,NFU1,AAK1,LOC787229,LOC104973411, ANXA4, GMCL1, SNRNP27,MXD1, ASPRV1, LOC104973412, LOC101905499, PCBP1,LOC107132938, C11H2orf42, TIA1, PCYOX1,LOC107132939,SNRPG,**EHD3, CAPN14**, GALNT14, CAPN13, **LCLAT1**, LOC101905676, **LBH**,YPEL5, LOC101905873, LOC104968430, ALK, CLIP4, C11H2orf71,FAM179A, LOC107132940, WDR43, TRMT61B, SPDYA,PPP1CB, PLB1 | | Fat deposition, high altiduted adaption, immune response, meat quality |
|  | 55 | |  | ROH island 0.995th per-centile | 26 | 21,539,987 -  22,954,453 | |  | LOC104975965, PAX2, SLF2, SEMA4G, MRPL43, C26H10orf2, LZTS2,PDZD7, SFXN3, KAZALD1, TLX1, LOC107131879, **LBX1**,LOC100847491, **BTRC**, LOC104975969, LOC783067, POLL, DPCD,FBXW4, LOC101908075, **FGF8** NPM3**, MGEA5**, KCNIP2, C26H10orf76,HPS6, LOC104975987, LDB1, PPRC1, NOLC1, LOC101902227,LOC785229, ELOVL3, PITX3, **GBF1** | | Leg confirmation, lipid metabolism, milk protein, and fat |

^1^di-value >10, ^2^ top candidate selection signatures which are 1 Mb up- or downstream of the middle position
