# Supplementary material for: Analyzing Runs of Homozygosity Reveals Patterns of Selection in German Brown Cattle
Source: Genes (Basel). 2024 Aug 9;15(8):1051. doi: 10.3390/genes15081051 (PMC11354284; doi:10.3390/genes15081051)
Supplement: Supplementary file 1 [file genes-15-01051-s001.zip › Supplementary Table S6.docx]

**Table S6.** Phenotypi**c** means, standard deviations (SD), and 75% confidence intervals (75% CI) of herd life (HL), length of productive life (LPL), number of calvings (NC), lifetime milk yield (LMY), lifetime fat yield (LFY), lifetime protein yield (LPY), effective lifetime milk yield (EffLMY), effective lifetime fat yield (EffLFY), effective lifetime protein yield (EffLPY), survival to 2nd (Surv1), 4th (Surv3), 6th (Surv5), 8th (Surv7), and 10^th^ (Surv9) lactation, culling rate due to claw and leg disorders (Cul_CL_), infertility (Cul_INF_), and udder diseases (Cul_UD_), proportion of US Brown Swiss and inbreeding coefficients in German Brown cows from all herds (no of cows=1,032,901) and the 258 herds randomly selected (no of cows=71,633) by the Allgäuer Herdebuchgesellschaft.

| **Trait** | **All herds (n=11,495)** | | | **Selected 258 herds** | | |
| --- | --- | --- | --- | --- | --- | --- |
|  | **Mean** | **SD** | **75% CI** | **Mean** | **SD** | **75% CI** |
| HL (years) | 5.98 | 2.57 | 3.91-7.57 | 6.11 | 2.81 | 3.82-7.87 |
| LPL (years) | 3.41 | 2.44 | 1.15-4.89 | 3.58 | 2.67 | 1.07-5.21 |
| NC | 3.32 | 2.25 | 1-5 | 3.53 | 2.44 | 1-5 |
| LMY (kg) | 22,662 | 18674 | 7663-33,300 | 26,140 | 22,096 | 8099-39,057 |
| LFY (kg) | 952 | 784 | 322-1400 | 1102 | 932 | 342-1644 |
| LPY (kg) | 812 | 672 | 273-1194 | 950 | 804 | 293-1421 |
| EffLMY (kg/day) | 8.841 | 4.638 | 5.312-12.159 | 9.794 | 5.279 | 5.737-13.605 |
| EffLFY (kg/day) | 0.372 | 0.197 | 0.223-0.512 | 0.413 | 0.225 | 0.241-0.574 |
| EffLPY (kg/day) | 0.317 | 0.170 | 0.188-0.437 | 0.356 | 0.195 | 0.206-0.496 |
| Surv1 | 0.760 |  |  | 0.773 |  |  |
| Surv3 | 0.423 |  |  | 0.465 |  |  |
| Surv5 | 0.184 |  |  | 0.229 |  |  |
| Surv7 | 0.059 |  |  | 0.086 |  |  |
| Surv9 | 0.014 |  |  | 0.024 |  |  |
| Cul_CL_ | 0.101 |  |  | 0.090 |  |  |
| Cul_INF_ | 0.231 |  |  | 0.214 |  |  |
| Cul_UD_ | 0.117 |  |  | 0.111 |  |  |
| US Brown Swiss (%) | 71.71 | 11.86 | 66-80 | 73.82 | 10.70 | 69-81 |
| F_Ped_ | 0.0244 | 0.0205 | 0.0105-0.0342 | 0.0279 | 0.0209 | 0.0143-0.0382 |
| F_a_Bal_ | 0.0508 | 0.0393 | 0.0190-0.0762 | 0.0632 | 0.0423 | 0.0283-0.093 |
| Ahc | 0.0541 | 0.0436 | 0.0193-0.0808 | 0.0679 | 0.0474 | 0.0290-0.1007 |
| F_a_Kal_ | 0.0051 | 0.0059 | 0.0006-0.0076 | 0.0068 | 0.0067 | 0.0014-0.0103 |
| F_New_ | 0.0200 | 0.0168 | 0.0092-0.0270 | 0.0221 | 0.0166 | 0.0120-0.0290 |
