# Supplementary material for: Analyzing Runs of Homozygosity Reveals Patterns of Selection in German Brown Cattle
Source: Genes (Basel). 2024 Aug 9;15(8):1051. doi: 10.3390/genes15081051 (PMC11354284; doi:10.3390/genes15081051)
Supplement: Supplementary file 1 [file genes-15-01051-s001.zip › Supplementary Table S7.docx]

**Table S7.** Inbreeding coefficient F_ROH_ based on different settings for ROH-definitions (The line in bold letters corresponds to the selected settings for this study).

| MAF | HWE | Min. length (kb) | Min. number of SNPs | Min. density (SNP/kb) | Max. gap (kb) | Window size  (SNP) | Max. number of het. SNPs | Missing SNPs | F_ROH_ |
| --- | --- | --- | --- | --- | --- | --- | --- | --- | --- |
|  |  | 3191 | 64 | 1/100 | 1000 | 10 | 1 | 0 | 0.1290 |
|  |  | 3191 | 64 | 1/100 | 1000 | 15 | 1 | 0 | 0.1253 |
|  |  | 3191 | 64 | 1/100 | 1000 | 20 | 1 | 0 | 0.1237 |
|  |  | 3191 | 64 | 1/100 | 1000 | 30 | 1 | 0 | 0.1198 |
|  |  | 3191 | 64 | 1/100 | 500 | 10 | 1 | 0 | 0.1252 |
|  |  | **3191** | **64** | **1/100** | **500** | **15** | **1** | **0** | **0.1215** |
|  |  | 3191 | 64 | 1/100 | 500 | 20 | 1 | 0 | 0.1200 |
|  |  | 3191 | 64 | 1/100 | 500 | 30 | 1 | 0 | 0.1163 |
| 0.01 | 0.0000001 | 3191 | 64 | 1/100 | 500 | 15 | 1 | 0 | 0.1077 |
|  |  | 1000 | 64 | 1/100 | 500 | 20 | 1 | 0 | 0.2244 |
| 0.01 | 0.0000001 | 1000 | 15 | 1/100 | 500 | 15 | 1 | 0 | 0.1946 |
| 0.01 |  | 2000 | 60 | 1/100 | 500 | 20 | 1 | 2 | 0.1130 |
|  |  | 2000 | 60 | 1/100 | 500 | 20 | 1 | 2 | 0.1269 |
|  |  | default | 50 | 1/100 | 1800 | default | 0 | 2 | 0.1188 |
| 0.01 | 0.0000001 | 1000 | 15 | 1/100 | 500 | default | 1 | 0 | 0.1154 |

The line in bold letters corresponds to the selected settings for this study.
