# Supplementary material for: Analyzing Runs of Homozygosity Reveals Patterns of Selection in German Brown Cattle
Source: Genes (Basel). 2024 Aug 9;15(8):1051. doi: 10.3390/genes15081051 (PMC11354284; doi:10.3390/genes15081051)
Supplement: Supplementary file 1 [file genes-15-01051-s001.zip › Supplementary Table S9.docx]

**Table S9.** Parameters for the detection of ROH islands used in previous studies on dairy cattle.

| **Reference** | **C** | **Number of SNPs** | **Imp** | **CR** | **MAF** | **HWE** | **Min. length (Mb)** | **Min. number of SNPs** | | **Min. density (SNP/kb)** | **Max. gap (Mb)** | **Window size** | **Max. number of het. SNPs** | **Missing SNPs** |
| --- | --- | --- | --- | --- | --- | --- | --- | --- | --- | --- | --- | --- | --- | --- |
| Ablondi et al. (2022) | I | 84,443 | Y | 95 | 0.01 | 0.005 | 1 | 15 | | 1/100 | 0.5 |  | 1 | 1 |
| Addo et al. (2019) | G | 40,851 | N | 95 | 0.05 | 0.0001 |  |  | | 1/100 | 1 | 50 | 1 | 2 |
| Cesarani et al. (2018) | I | 40,099 | N | 97.5 | 0.02 | 0.01 | 1 | 15 | |  |  |  | 0 | 0 |
| (Doekes et al., 2018) | NL | 75,538 | Y | 85 | 0.025 |  | 3.75 | | 38 |  | 0.5 |  | 0 |  |
| Ferenčaković et al. (2013) | AUT | 36,273 | N | 95 | 0.01 |  | 1 | | 15 |  | 1 |  | 0 | 5 |
| Lozada-Soto et al. (2022) | US | 62,464 | Y | 95 | 0.01 |  | 2 | | 60 | 1/100 | 0.5 |  | 1 | 2 |
| Makanjuola et al. (2020) |  | 43,126 | Y | 95 | 0.01 |  | 1 | | 20 | 1/100 | 0.5 |  |  |  |
| Marras et al. (2015) | I | 44,325 | N | 97.5 | 0.01 | - | 1 | | 15 |  |  |  | 0 | 0 |
| Moscarelli et al. (2021) | I | 34,735 | N | 95 | 0.01 |  | 1 | | 30 | 1/1000 |  |  | 1 | 1 |
| Purfield et al. (2012) |  | 43,028 | N | 90 |  | 0.0001 | 0.5 | | 58 | 1/50 | 0.1 |  | 1 | 2 |
| Purfield et al. (2012) |  | 43,028 | N | 90 |  | 0.0001 | 0.5 | |  | 1/120 | 1 |  | 1 | 2 |
| Schiavo et al. (2022) |  | 130.746 | N | 90 | - | 0.0001 | 1 | | 15 | 1/100 | 1 |  | 2 |  |
| Signer-Hasler et al. (2017) | CH | 27,612 | N |  | 0.01 | 0.0001 |  | | 50 | 1/100 | 1.8 |  | 0 | 2 |
| Szmatoła et al. (2019) | POL | 42,717 |  | 95 |  |  |  | | 30 |  | 1 |  | Different categories:  >16: 1  Others: 0 | 4,8: 1  8,16: 2  >16: 4 |
| Visser et al. (2023) | South Africa | 35,572 |  | 95 | - | - | 1 | | 54 | 1/75 | 0.5 |  | 0 | 2 |
| Zhang et al. (2015) |  |  |  |  |  |  | 0.01 | | 20 | 1/1000 |  |  | 1 |  |

C= Country; CR= call rate of SNPs; MAF= minor allele frequency; HWE=Hardy-Weinberg-Equilibrium; Y = Yes, N = No; Imp = imputed genotype data; Min. length=minimum length of hom

[10] Ablondi, M., A. Sabbioni, G. Stocco, C. Cipolat-Gotet, C. Dadousis, J.-T. van Kaam, R. Finocchiaro und A. Summer, (2022): Genetic Diversity in the Italian Holstein Dairy Cattle Based on Pedigree and SNP Data Prior and After Genomic Selection. Frontiers in veterinary science **8**, 773985-773985.

[11] Addo, S., S. Klingel, D. Hinrichs und G. Thaller, (2019): Runs of Homozygosity and NetView analyses provide new insight into the genome-wide diversity and admixture of three German cattle breeds. PLOS ONE **14**, e0225847.

[19] Cesarani, A., S. Sorbolini, A. Criscione, S. Bordonaro, G. Pulina, G. Battacone, D. Marletta, G. Gaspa und N. Macciotta, (2018): Genome‐wide variability and selection signatures in Italian island cattle breeds. Anim. Genet. **49**, 371-383.

[46] Doekes, H. P., R. F. Veerkamp, P. Bijma, S. J. Hiemstra und J. J. Windig, (2018): Trends in genome-wide and region-specific genetic diversity in the Dutch-Flemish Holstein–Friesian breeding program from 1986 to 2015. Genetics Selection Evolution **50**, 15.

[15] Ferenčaković, M., E. Hamzić, B. Gredler, T. Solberg, G. Klemetsdal, I. Curik und J. Sölkner, (2013): Estimates of autozygosity derived from runs of homozygosity: empirical evidence from selected cattle populations. J. Anim. Breed. Genet. **130**, 286-293.

[52] Lozada-Soto, E. A., F. Tiezzi, J. Jiang, J. B. Cole, P. M. VanRaden und C. Maltecca, (2022): Genomic characterization of autozygosity and recent inbreeding trends in all major breeds of US dairy cattle. J. Dairy Sci. **105**, 8956-8971.

[51] Makanjuola, B. O., C. Maltecca, F. Miglior, F. S. Schenkel und C. F. Baes, (2020): Effect of recent and ancient inbreeding on production and fertility traits in Canadian Holsteins. BMC genomics **21**, 1-15.

[50] Marras, G., G. Gaspa, S. Sorbolini, C. Dimauro, P. Ajmone‐Marsan, A. Valentini, J. L. Williams und N. P. Macciotta, (2015): Analysis of runs of homozygosity and their relationship with inbreeding in five cattle breeds farmed in Italy. Anim. Genet. **46**, 110-121.

[18] Moscarelli, A., M. T. Sardina, M. Cassandro, E. Ciani, F. Pilla, G. Senczuck, B. Portolano und S. Mastrangelo, (2021): Genome-wide assessment of diversity and differentiation between original and modern Brown cattle populations. Anim. Genet. **52**, 21-31.

[7] Purfield, D. C., D. P. Berry, S. McParland und D. G. Bradley, (2012): Runs of homozygosity and population history in cattle. BMC Genetics **13**, 70.

[13] Schiavo, G., S. Bovo, A. Ribani, G. Moscatelli, M. Bonacini, M. Prandi, E. Mancin, R. Mantovani, S. Dall'Olio und L. Fontanesi, (2022): Comparative analysis of inbreeding parameters and runs of homozygosity islands in 2 Italian autochthonous cattle breeds mainly raised in the Parmigiano-Reggiano cheese production region. J. Dairy Sci. **105**, 2408-2425.

[29] Signer-Hasler, H., A. Burren, M. Neuditschko, M. Frischknecht, D. Garrick, C. Stricker, B. Gredler, B. Bapst und C. Flury, (2017): Population structure and genomic inbreeding in nine Swiss dairy cattle populations. Genet Sel Evol **49**, 83.

[72] Szmatoła, T., A. Gurgul, I. Jasielczuk, T. Ząbek, K. Ropka-Molik, Z. Litwińczuk und M. Bugno-Poniewierska, (2019): A Comprehensive Analysis of Runs of Homozygosity of Eleven Cattle Breeds Representing Different Production Types. Animals **9**, 1024.

[14] Visser, C., S. F. Lashmar, J. Reding, D. P. Berry und E. van Marle-Köster, (2023): Pedigree and genome-based patterns of homozygosity in the South African Ayrshire, Holstein, and Jersey breeds. Frontiers in Genetics **14**.

[16] Zhang, Q., M. P. L. Calus, B. Guldbrandtsen, M. S. Lund und G. Sahana, (2015): Estimation of inbreeding using pedigree, 50k SNP chip genotypes and full sequence data in three cattle breeds. BMC Genetics **16**, 88.
