# Supplementary figures and images for: Analyzing Runs of Homozygosity Reveals Patterns of Selection in German Brown Cattle
Source: Genes (Basel). 2024 Aug 9;15(8):1051. doi: 10.3390/genes15081051 (PMC11354284; doi:10.3390/genes15081051)

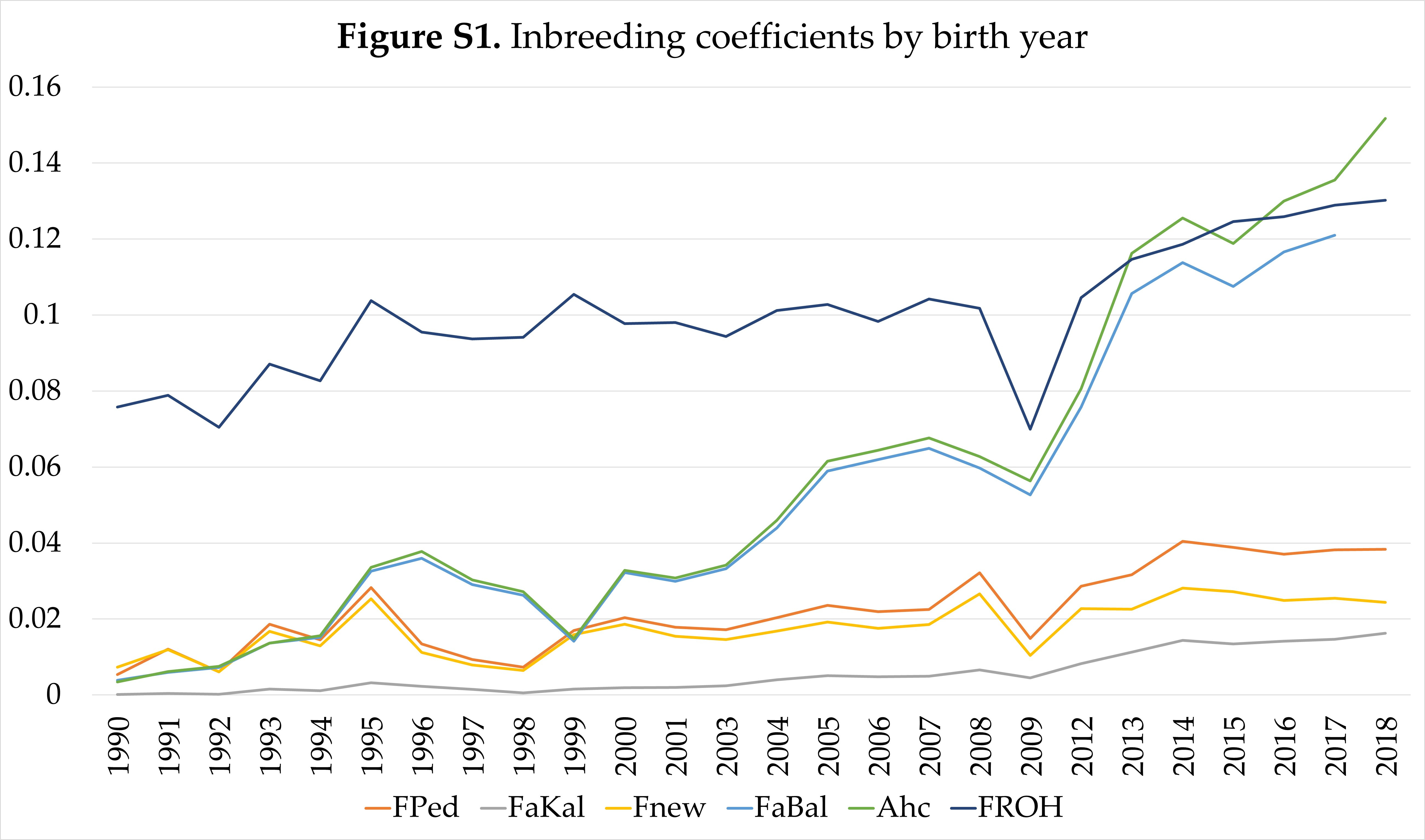

Supplement: Supplementary file 1 [file genes-15-01051-s001.zip › Supplementary FigureS1-rev.jpg]

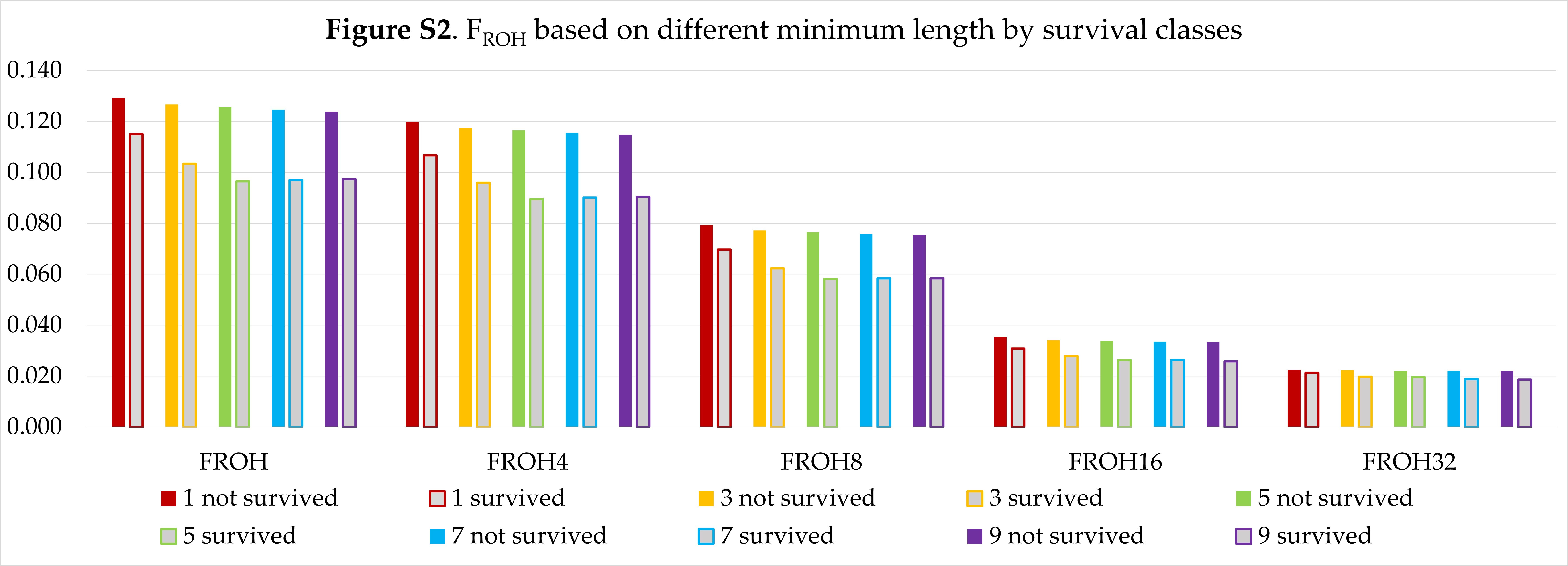

Supplement: Supplementary file 1 [file genes-15-01051-s001.zip › Supplementary FigureS2-rev.jpg]
